# Evaluating the effects of tDCS on depressive and anxiety symptoms from a transdiagnostic perspective: a systematic review and meta-analysis of randomized controlled trials

**DOI:** 10.1038/s41398-024-03003-w

**Published:** 2024-07-18

**Authors:** Esther Zhiwei Zheng, Nichol M. L. Wong, Angela S. Y. Yang, Tatia M. C. Lee

**Affiliations:** 1 State Key Laboratory of Brain and Cognitive Sciences, The University of Hong Kong, Pok Fu Lam, Hong Kong; 2 Laboratory of Neuropsychology & Human Neuroscience, The University of Hong Kong, Pok Fu Lam, Hong Kong; 3 Department of Psychology, The Education University of Hong Kong, Ting Kok, Hong Kong

**Keywords:** Depression, Human behaviour

## Abstract

Depressive and anxiety symptoms are prevalent among patients with various clinical conditions, resulting in diminished emotional well-being and impaired daily functioning. The neural mechanisms underlying these symptoms, particularly across different disorders, remain unclear, limiting the effectiveness of conventional treatments. Therefore, it is crucial to elucidate the neural underpinnings of depressive and anxiety symptoms and investigate novel, effective treatments across clinical conditions. Transcranial direct current stimulation (tDCS) is a neuromodulatory technique that can help understand the neural underpinnings of symptoms and facilitate the development of interventions, addressing the two research gaps at both neural and clinical levels. Thus, this systematic review and meta-analysis aims to evaluate the existing evidence regarding the therapeutic efficacy of tDCS in reducing depressive and anxiety symptoms among individuals with diverse clinical diagnoses. This review evaluated evidence from fifty-six randomized, sham-controlled trials that administered repeated tDCS sessions with a parallel design, applying a three-level meta-analytic model. tDCS targeting the left dorsolateral prefrontal cortex (DLPFC) at 2-mA intensity demonstrates moderate efficacy in alleviating depressive symptoms, identifying the left DLPFC as a transdiagnostic neural mechanism of depressive symptoms across clinical conditions. In comparison, the findings on anxiety symptoms demonstrate greater heterogeneity. tDCS over the left DLPFC is effective in reducing depressive symptoms and shows promising effects in alleviating anxiety symptoms among individuals with diverse diagnoses. These findings enhance our understanding of the neuropsychological basis of depressive and anxiety symptoms, laying the groundwork for the development of more effective tDCS interventions applicable across clinical conditions.

## Introduction

Depressive and anxiety symptoms are highly prevalent across various clinical conditions and often co-occur, negatively impacting disease prognoses and quality of life [1–3]. The neural underpinnings of these two symptoms across clinical disorders and the co-occurrence of these two symptom categories are unclear [4–6], potentially contributing to the lack of effective treatments [7, 8]. Indeed, a significant portion of patients experiencing these symptoms do not respond well to conventional treatments [9, 10]. Therefore, it is important to understand how these two symptoms can be targeted across clinical conditions.

Neuromodulation therapies, such as Transcranial Magnetic Stimulation (TMS), Transcranial Alternating Current Stimulation (tACS), and Transcranial Direct Current Stimulation (tDCS), offer targeted, non-pharmacological options for modulating brain activity, presenting personalized treatment potentials with reduced side effects for neurological and psychiatric disorders [11]. tDCS can be one approach for furthering the understanding of depressive and anxiety symptoms across conditions and one possible treatment for alleviating these two symptoms across disorders due to its several advantages. It is a non-invasive neuromodulatory technique that modulates neuronal excitability by delivering weak electric current (1–2 mA) to the targeted brain regions [12, 13]. As a substantial proportion of patients have shown poor responses to traditional treatments of psychiatric disorders [3, 14, 15], tDCS is a safe, well-tolerated tool that has been increasingly studied as an alternative or add-on treatment [16–21]. In addition to being a safe treatment option, tDCS can directly target the altered neural activity and functional connectivity of brain regions underlying these symptoms, potentially through mechanisms such as the modulation of neuronal membrane potential, strengthening synaptic connections through long-term potentiation, and enhancing levels of brain-derived neurotrophic factor (BDNF) [22–24]. Thus, tDCS can help establish causal relationships between brain regions and targeted symptoms, thus elucidating the symptoms’ underlying neural underpinnings.

Existing evidence has consistently supported the effectiveness of tDCS interventions for psychiatric disorders. Numerous studies and reviews have provided a high level of evidence demonstrating the therapeutic efficacy of tDCS specifically in Major Depressive Disorder (MDD) [25–29]. According to evidence-based, meta-analytic guidelines [27, 28], repeated tDCS targeting the left dorsolateral prefrontal cortex (DLPFC, F3) has been found to be effective in alleviating depressive symptoms in MDD patients. Furthermore, although the evidence is not as robust, tDCS has also shown promising results in the treatment of anxiety disorders such as Generalized Anxiety Disorder (GAD) and Social Anxiety Disorder (SAD) [20, 30]. These findings highlight tDCS interventions as a safe, cost-effective, and viable treatment option for individuals diagnosed with MDD and anxiety disorders.

However, existing studies and reviews have only focused on evaluating the therapeutic efficacy of tDCS on depressive and anxiety symptoms in patients diagnosed with MDD and anxiety disorders. No review has systematically evaluated evidence on the effects of tDCS on depressive and anxiety symptoms across clinical conditions. As depressive and anxiety symptoms are not limited to MDD and anxiety disorders [31, 32], it is crucial to examine effective treatments for alleviating depressive and anxiety symptoms across heterogeneous clinical populations. This can potentially improve the treatment options and emotional well-being of patients who suffer from these two affective symptoms regardless of their primary diagnoses. Additionally, as these two affective symptoms are often comorbid [33], a better understanding of the shared neural underpinnings of these two symptoms would be necessary for developing more effective treatments. As a result, evaluating evidence on the therapeutic efficacy of tDCS in treating depressive and anxiety symptoms in patients with various clinical diagnoses is essential for addressing this gap in clinical treatments and contributing to a better understanding of the neural underpinnings of these two prevalent affective symptoms.

This review aims to fill the research gap by evaluating evidence from randomized controlled trials (RCTs) that administered repeated (more than one session, without restrictions on the interval between sessions) tDCS sessions with a parallel design. The focus is on evidence with high methodological quality, as RCTs can reduce bias at each study phase and better investigate treatment effects [34]. Repeated stimulation has the potential for cumulative tDCS effects [35] and has demonstrated therapeutic efficacy in clinical populations [36, 37]. Additionally, a parallel design allows for better examination of treatment outcomes by controlling for potential carryover effects [38]. Lastly, conventional tDCS is specifically evaluated as conventional tDCS and high-definition tDCS (HD-tDCS) have distinct protocols and thus can demonstrate neuromodulatory effects through different mechanisms of action [39].

In summary, this systematic review and meta-analysis aim to synthesize and quantitively evaluate the effects of tDCS in alleviating depressive and anxiety symptoms across various clinical conditions. The review focuses on evidence from RCTs that administered repeated, conventional tDCS with a parallel design, with a primary goal of identifying potential transdiagnostic neural underpinnings and exploring effective tDCS interventions for depressive and anxiety symptoms across various clinical populations.

## Methods

This systematic review was not pre-registered, and no protocol can be accessed. However, the Preferred Reporting Items for Systematic Reviews and Meta-Analysis (PRISMA) guidelines were strictly followed [40]. The review objectives, screening criteria and strategy, study selection and data extraction procedures, risk of bias assessment, and anticipated completion date were outlined prior to commencing the review process. Additionally, thorough efforts were made to ensure there was no existing review on the same topic. The PRISMA checklist can be found in supplementary materials.

### Inclusion and exclusion criteria

The inclusion criteria for selecting studies were: (a) papers written in English; (b) studies on human subjects; (c) original empirical research on conventional tDCS; (d) randomized, sham-controlled trials with a parallel design; (e) studies with affective outcomes or clinical conditions demonstrating affective symptoms; (f) studies administered more than one tDCS sessions.

Studies were excluded when they satisfied one of the following criteria: (a) review papers; (b) abstracts or conference proceedings; (c) study protocols; (d) studies unrelated to tDCS; (e) studies on HD-tDCS; (f) studies examined tDCS combined with other interventions, treatments, or training; (g) studies with duplicate samples.

### Searching strategies

**Fig. 1:**
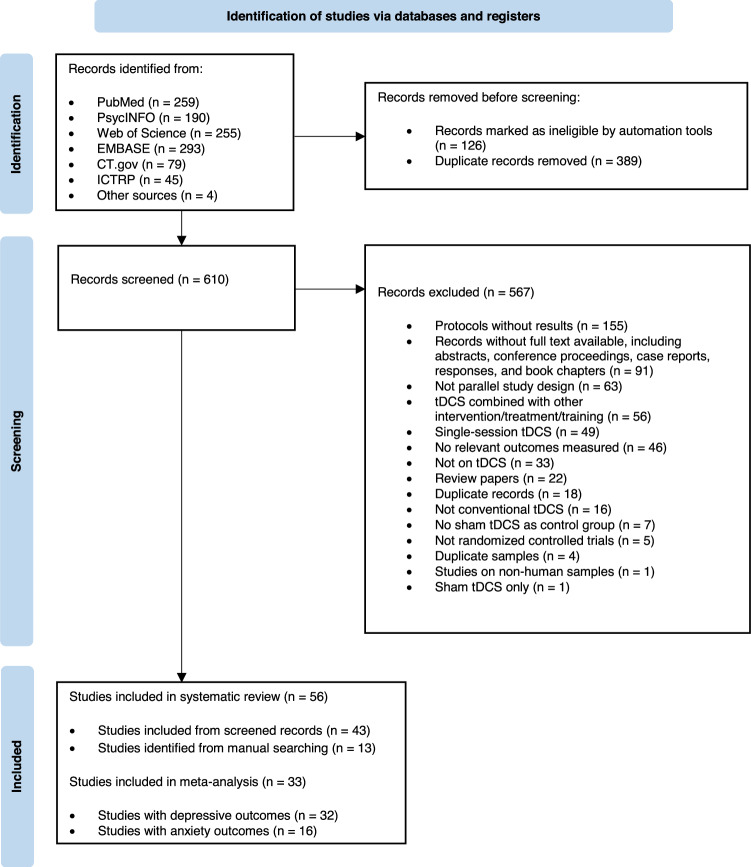
PRISMA flow diagram of study selection.

Databases and registers, including PubMed, PsycINFO, Web of Science, EMBASE, ClinicalTrials.gov (CT.gov), and International Clinical Trials Registry Platform (ICTRP), were searched on March 20, 2024. The search string used was: (tDCS OR transcranial direct current stimulation) AND (emotion* OR affective* OR mood*) AND (sham* OR placebo*) AND (random*). This search string was adopted to generate a more comprehensive list of studies examining affective outcomes (including depressive and anxiety symptoms) without overlooking papers that might meet the criteria. Records generated from the search results were combined and imported to EndNote for duplicate removal. Two authors (EZZ, ASYY) independently screened the remaining records based on the inclusion and exclusion criteria by reading their titles, abstracts, and main texts. The review papers identified from the initial records were retrieved for screening of their references. Screening result discrepancies were resolved through consensus discussions (Fig. 1).

### Data extraction

Data extraction was performed together by the two authors (EZZ, ASYY). Variables extracted included study and participant characteristics (e.g., participants’ primary diagnoses as indicated in the original studies), tDCS protocol-related information (e.g., montage), outcome measures, and the main results indicating treatment efficacy. A consensus on the accuracy of the extracted information was reached through discussions.

### Meta-analysis

All analyses were performed using the metafor package [41] within the statistical software environment R. The effects of tDCS on depressive and anxiety symptoms were evaluated separately. Due to some studies including multiple measurements for these symptoms, a three-level structure was applied to the meta-analytic model to account for between-study and within-study heterogeneity and the dependence of effect sizes [42–46].

The endpoint means and standard deviations of depressive and anxiety outcomes were extracted for the active and sham groups. If the outcome data were unavailable, the corresponding authors were contacted systematically. The standardized mean difference (SMD) was then calculated to report the intervention effect, considering the different measurements used in the included studies. To assess potential publication bias, funnel plots, and Egger’s regression test were applied [47, 48]. In cases where significant publication bias was detected, leave-one-out analysis was conducted. Additionally, moderator analyses were conducted to examine the moderating effects of stimulation intensity and duration (coded as dummy variables), in instances of significant heterogeneity.

### Risk of bias assessment

The Cochrane Collaboration’s risk-of-bias tool was applied to assess the methodological quality of the studies [49]. Two authors (EZZ, ASYY) independently rated the risk of bias (ROB) in each study in domains including random sequence generation, allocation concealment, blinding of participants and personnel, blinding of outcome assessment, incomplete outcome data, and selective outcome reporting by giving a rating of low, unclear, or high ROB. Each study’s overall ROB was given based on the criteria used in a meta-analytic systematic review of tDCS and MDD [50]. Discrepancies in the ratings were resolved through discussions.

## Results

### Study selection

A total of 1125 records were identified from the initial search (259 from PubMed, 190 from PsycINFO, 255 from Web of Science, 293 from EMBASE, 79 from CT.gov, 45 from ICTRP, and 4 from other sources). After applying filters, 126 records were excluded. After importing the remaining records in EndNote, 389 duplicate records were removed. The remaining 610 records were screened by examining their titles, abstracts, and full texts based on the pre-specified criteria, and 567 records were excluded. A total of 43 studies were included after screening the initial search records. The references of the review papers identified from the initial search were screened, resulting in 13 more studies. In summary, 56 studies were included in the systematic review; 33 of these studies with available data were included in the meta-analysis (Fig. 1).

### Study overview

Table 1 provides comprehensive information on sample characteristics, tDCS protocols, and measurements of depressive and anxiety symptoms across the 56 studies published between 2006 and 2024. The combined participant count across these studies was 2349, with sample sizes ranging from 10 to 174. The most frequently implemented tDCS protocol involved targeting the left DLPFC as the anodal stimulation site, employing an intensity of 2 mA, and administering stimulation for 20 min per session. Among the included studies, 43 investigated the effects of tDCS interventions on depressive symptoms, and 24 examined anxiety symptoms.

**Table 1:** Overview of included studies in alphabetical order.

Study	Sample characteristics				tDCS protocols			Symptom measurement	
	Diagnosis	N (Total)	% Female	Age (SD)	Montage (anode, cathode)	Intensity (mA)	Duration (min)	Depressive symptoms	Anxiety symptoms
Acler et al. [94]	Post-polio syndrome	32	53%	61.4 (5.9)	Right and left pre-motor cortex (2 cm ahead C3-C4), Left shoulder	1.5	15	HDRS	–
Ahmadizadeh et al. [68]	PTSD	40	65%	43.8 (10.6)	F3, F4	2	20	BDI-II	BAI
Aksu et al. [69]	Panic disorder	30	63%	37.3 (11.5)	F3, F4	2	20	HDRS; BDI	HARS; PDSS
Barham et al. [100]	ADHD	22	68%	22.0 (2.8)	F4, F3	2	20	–	–
Bennabi et al. [51]	Treatment-resistant MDD	24	75%	61.8 (16.3)	F3, Fp2	2	30	MADRS; HDRS; BDI	STAI
Benninger et al. [95]	Parkinson’s disease	25	36%	NR	Symmetrically either over the premotor and motor (electrode center 10 mm anterior to Cz) or prefrontal cortices (forehead above eyebrows), Mastoids	2	20	BDI	–
Blumberger et al. [52]	Treatment-resistant MDD	24	83%	47.3; Range: 24–62	F3, F4	2	20	MADRS; HDRS; BDI	–
Boggio et al. [53]	MDD	40	70%	49.4 (7.4)	F3, Fp2	2	20	HDRS; BDI	–
Borckardt et al. [83]	Patients undergoing unilateral primary TKA	39	74%	67.0 (9.1)	Knee representation of the motor strip, F4	2	20	–	–
Brunelin et al. [80]	Schizophrenia with verbal hallucinations	30	NR	NR	Left DLPFC (Midway FP1/F3), Left TPJ (Midway T3/P3)	2	20	–	–
Chen et al. [65]	MDD	63	71%	NR	F3, Fp2	2	60 30	HDRS	HARS
da Silva et al. [91]	Lesch’s type IV alcohol-dependent patients	13	0%	49.0 (29.6)	F3, Right supradeltoid area	2	20	HDRS	HARS
de Lima et al. [70]	GAD	30	63%	NR	F3, Fp2	2	20	BDI	HARS; BAI
Doruk et al. [96]	Parkinson’s disease	18	33%	61 (8); Range: 40–71	F3, Fp2 F4, Fp1	2 2	20 20	BDI; HDRS	HARS
Dutra et al. [84]	Primary dysmenorrhea	26	100%	NR	F3, Fp2	2	20	–	HARS
Fitzgerald et al. [78]	Schizophrenia or schizoaffective disorder	13	NR	NR	F3, TP3 F3 and F4, TP3 and TP4	2 2	20 20	CDSS	–
Fregni et al. [54]	MDD	10	NR	42.7 (10)	F3, Fp2	1	20	HDRS; BDI	–
Fregni et al. [55]	MDD	18	61%	46.4 (9.4)	F3, Fp2	1	20	HDRS	–
Fregni et al. [85]	Fibromyalgia	32	100%	53.4 (8.9)	C3, Fp2 F3, Fp2	2 2	20 20	BDI	VAS - Anxiety
Huang et al. [66]	Unipolar and bipolar depression	70	74%	NR	Fp1, Fp2 F3, F4	2 2	20 20	MADRS; HDRS; QIDS-SR	HARS
Jafari et al. [71]	SAD	45	44%	32.4 (7.0)	F3, Medial PFC F3, Medial PFC	1 2	20 20	BDI-II	LSAS; PSWQ
Jeon et al. [74]	Schizophrenia	54	52%	NR	F3, F4	2	30	CDSS	–
Khedr et al. [86]	Fibromyalgia	36	94%	NR	C3, Right arm	2	20	HDRS	HARS
Klírová et al. [106]	Post-COVID syndrome	33	70%	NR	F3, F4	2	30	PHQ-9	GAD-7
Koops et al. [81]	Medication-resistant auditory hallucinations	54	54%	Range: 23–74	Left DLPFC (Midway FP1/F3), left TPJ (Midway T3/P3)	2	20	–	–
Leffa et al. [101]	ADHD	64	47%	38.3 (9.6)	F4, F3	2	30	BDI	BAI
Lisoni et al. [103]	BPD	30	60%	40.3 (12.8)	F4, F3	2	20	HDRS; BDI	HARS; IDAS – Anxiety subitem
Lisoni et al. [82]	Schizophrenia	50	22%	42.70 (12.11)	F3, Fp2	2	20	CDSS	–
Liu et al. [97]	Well-controlled temporal lobe epilepsy	33	42%	NR	F3, Fp2	2	20	BDI-II; NDDI-E	–
Loo et al. [58]	MDD	40	55%	NR	F3, F8	1	20	MADRS; HDRS; BDI	–
Loo et al. [56]	MDD	60	47%	NR	F3, F8	2	20	MADRS	–
Loo et al. [57]	MDD; bipolar depression	84	50%	NR	F3, F8	2.5	30	MADRS	–
Maas et al. [98]	Spinocerebellar ataxia type 3	20	40%	NR	Cerebellum, right deltoid muscle	2	20	PHQ-9	–
Mariano et al., 2019	Chronic low back pain	21	14%	63.1 (10.5)	Right mastoid process, FC1	2	20	PHQ-9	GAD-7; PASS-20
Molavi et al. [104]	BPD	32	47%	30.6 (5.4)	F3, F4	2	20	BDI	–
Mondino et al., 2016	Schizophrenia	23	35%	NR	Midway F3 and Fp1 (left DLPFC), Midway T3 and P3 (left TPJ)	2	20	–	–
Newstead et al. [72]	Healthy individuals	21	52%	NR	F3, Right cerebellum	2	12	–	–
Palm et al. [76]	Schizophrenia	20	25%	36.1 (11.4)	F3, Fp2	2	20	CDSS	–
Pegado et al., 2020	Primary dysmenorrhea	20	100%	NR	F3, Right supradeltoid area	2	20	–	HARS
Pinto et al. [105]	Primary Sjogren syndrome	36	100%	NR	F4, F3	2	20	–	VAS - Anxiety
Pinto et al. [99]	Stroke	60	27%	Range: 21–67	Primary motor cortex (C3/C4) ipsilesional to the side of the stroke, Contralesional primary motor cortex (C3/C4)	Current ramped up across 30 to 60s to reach a target amplitude of between 2 and 3 mA	30	HDRS	HARS
Quintiliano et al. [89]	Chronic kidney disease patients with chronic pain	30	73%	NR	C3, Fp2	2	20	BDI	HARS
Salehinejad et al. [59]	MDD	30	57%	NR	F3, F4	2	20	HDRS; BDI	–
Salehinejad et al. [60]	MDD	24	63%	NR	F3, F4	2	20	HDRS; BDI	–
Samartin-Veiga et al. [90]	Fibromyalgia	130	100%	NR	C3, Fp2 F3, Fp2 OIC multi-electrode montage	2 2 Differed by electrode	20 20 20	HADS	HADS
Sampaio-Junior et al. [61]	Bipolar depression	59	68%	45.9 (12)	F3, F4	2	30	MADRS; HDRS	–
Shahbabaie et al. [92]	Early-abstinent methamphetamine users	90	0%	30.8 (6.2)	F3, Right shoulder F4, Left shoulder F3, Right supraorbital ridge F4, Left supraorbital ridge F3, F4	2 2 2 2 2	13 13 13 13 13	–	–
Sharafi et al. [62]	Treatment-resistant MDD	30	53%	47.2 (12.0)	F3, F4	2	20	HDRS	–
Silva et al. [73]	Treatment-resistant OCD	43	60%	NR	Left deltoid, SMA (1.5 cm anteriorly to the measured location of Cz)	2	30	BDI	BAI
Smith et al. [79]	Schizophrenia or schizoaffective disorder	33	27%	NR	F3, Fp2	2	20	–	–
Valiengo et al. [63]	Post-stroke depression	48	50%	NR	F3, F4	2	30	MADRS; HDRS	–
Valiengo et al. [77]	Schizophrenia	100	20%	35.5 (9.3)	Left DLPFC (Midway FP1/F3), left TPJ (Midway T3/P3)	2	20	CDSS	–
Verveer et al. [93]	Smokers	71	51%	22.0 (4.7)	F4, F3	2	13	–	–
Vigod et al. [64]	MDD	20	100%	32.3 (4.2)	F3, F4	2	30	MADRS; EPDS	STAI
Woodham et al. [67]	MDD	174	69%	NR	F3, F4	2	30	MADRS; HDRS	HARS
Zemestani et al. [102]	ASD	32	28%	10.2 (1.9)	F3, F4	1.5	15	–	–

Montage: C3, left M1; C4, right M1; F3, left DLPFC; F4, right DLPFC; F8, right orbit; FC1, left dorsal anterior cingulate cortex (dACC); Fp1, left supraorbital area; Fp2, right supraorbital area; OIC, left operculo-insular cortex; SMA, supplementary motor area; TP3/4, temporoparietal area; TPJ, temporoparietal junction.

Depressive Symptom Measurement: BDI, Beck Depression Inventory; CDSS, Calgary Depression Scale for Schizophrenia; EPDS, Edinburgh Postnatal Depression Scale; HADS, Hospital Anxiety and Depression Scale; HDRS, Hamilton Depression Rating Scale; MADRS, Montgomery–Åsberg Depression Rating Scale; NDDI-E, Neurological Disorders Depression Inventory for Epilepsy; PHQ-9, Patient Health Questionnaire; QIDS-SR, Quick Inventory of Depressive Symptomatology Self Report.

Anxiety Symptom Measurement: BAI, Beck Anxiety Inventory; GAD-7, Generalized Anxiety Disorder Scale; HARS, Hamilton Anxiety Rating Scale; IDAS, Irritability-Depression-Anxiety Scale; LSAS, Liebowitz Social Anxiety Scale; PASS, Pain Anxiety Symptoms Scale; PDSS, Panic Disorder Severity Scale; PSWQ, Penn State Worry Questionnaire; STAI, State-Trait Anxiety Inventory; VAS, Visual Analog Scale.

*ADHD* attention deficit hyperactivity disorder, *ASD* autism spectrum disorder, *BPD* borderline personality disorder, *GAD* generalized anxiety disorder, *MDD* major depressive disorder, *OCD* obsessive-compulsive disorder, *PTSD* posttraumatic stress disorder, *SAD* social anxiety disorder, *TKA* total knee arthroplasty, *NR* not reported.

Participant diagnoses varied in the studies. Seventeen studies focused on depression [51–67]. Four studies focused on anxiety disorders [68–71]. One study included healthy individuals [72]. Other diagnoses addressed in the studies encompassed Obsessive-Compulsive Disorder (OCD) [73], Schizophrenia or Schizoaffective Disorder [74–82], pain conditions [83–90], substance use [91–93], neurological disorders [94–99], Attention Deficit Hyperactivity Disorder (ADHD) [100, 101], Autism Spectrum Disorder (ASD) [102], Borderline Personality Disorder (BPD) [103, 104], Primary Sjogren Syndrome [105], and post-COVID syndrome [106].

Subsequent sections present the effects of tDCS on depressive symptoms, anxiety symptoms, and other affective outcomes. The results regarding tDCS effects are presented in terms of the comparison between the active and sham groups before and after interventions.

### tDCS effects on depressive symptoms

#### Overview

**Fig. 2:**
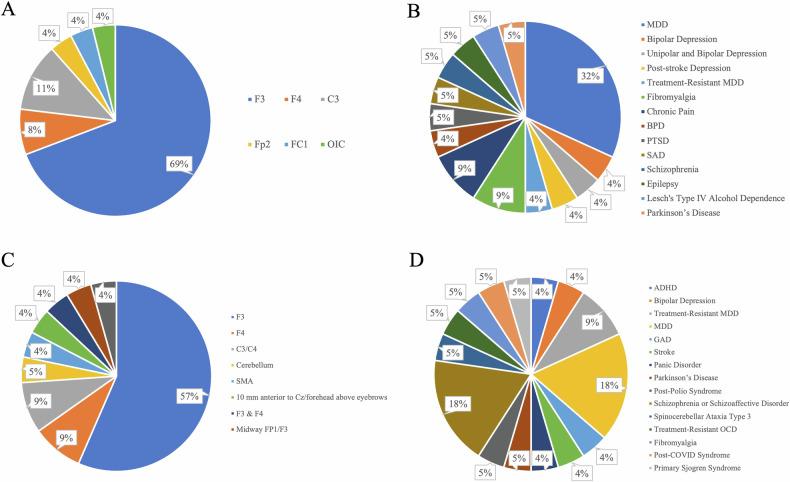
Breakdown of target regions and clinical conditions in studies with depressive outcomes. **A** Target regions in studies with depressive symptom alleviation; **B** Clinical conditions in studies with depressive symptom alleviation; **C** Target regions in studies without depressive symptom alleviation; **D** Clinical conditions in studies without depressive symptom alleviation. (Target region abbreviation: C3, left M1; C4, right M1; F3, left DLPFC; F4, right DLPFC; FC1, left dorsal anterior cingulate cortex (dACC); Fp1, left supraorbital area; Fp2, right supraorbital area; OIC, left operculo-insular cortex; SMA, supplementary motor area. Condition abbreviations: ADHD attention deficit hyperactivity disorder, BPD borderline personality disorder, GAD generalized anxiety disorder, MDD major depressive disorder, OCD obsessive-compulsive disorder, PTSD posttraumatic stress disorder, SAD social anxiety disorder).

Twenty-two studies showed positive effects of tDCS on depressive symptoms across various clinical conditions (Table S1; Fig. 2B). The left DLPFC was the target region for 18 of these studies (Table S1; Fig. 2A). Anodal stimulation over the left primary motor cortex (M1; C3) and the right DLPFC (F4) also showed positive effects. Twenty studies utilized an intensity of 2 mA. The stimulation duration consisted of a single session lasting either 20 or 30 min.

In contrast to studies that demonstrated positive effects of tDCS, the 21 studies that did not show positive effects exhibited greater heterogeneity in terms of the clinical diagnoses, stimulation protocols, sham conditions, and depressive symptom measurement (Table S1; Fig. 2C, D). The applied current intensity ranged from 1 to 2.5 mA, with 2 mA being the most frequently used intensity (17 studies). The duration of a single stimulation session ranged from 15 to 30 min.

#### Meta-analysis results: all studies

The multivariate meta-analysis model included 32 studies, encompassing 67 outcomes reflecting levels of depressive symptoms (Table 2; Fig. S1).

**Table 2:** Meta-analysis results on depressive and anxiety symptoms.

Studies	tDCS intervention effects					Heterogeneity		Egger’s regression test		Moderator:intensity		Moderator:duration	
	Estimate	t	*p*	SE	CI	Within-study variance	Between-study variance	t	*p*	F	*p*	F	*p*
**Studies with depressive outcomes** ***All studies*** *(study n* *=* *32, outcome n* *=* *67)*													
	–0.355	–4.049	<0.001***	0.088	[–0.530, –0.180]	Insignificant	Significant	–1.523	0.133	2.095	0.092	1.146	0.337
**Studies with depressive outcomes** ***F3 studies*** *(study n* *=* *23, outcome n* *=* *50)*													
	–0.422	–4.393	<0.001***	0.096	[–0.616, –0.229]	Insignificant	Significant	–1.756	0.085	3.696	0.032*	0.827	0.444
**Studies with anxiety outcomes** ***All studies*** *(study n* *=* *16, outcome n* *=* *27)*													
	–0.398	–2.049	0.051	0.194	[–0.796, 0.001]	Insignificant	Significant	–2.602	0.015*	0.483	0.698	2.767	0.083
**Studies with anxiety outcomes** ***F3 studies*** *(study n* = *9, outcome n* = *16)*													
	–0.449	–1.428	0.174	0.314	[–1.119, 0.221]	Insignificant	Significant	–1.806	0.093	1.801	0.201	1.287	0.309

*p < 0.05, **p < 0.01, ***p < 0.001.

The overall effect of active tDCS interventions on depressive symptoms was significant (estimate = –0.355, *t*(66) = –4.049, *p* < 0.001), with a standard error of 0.088. This indicated that the active interventions were associated with significantly lower levels of depressive symptoms compared to the sham intervention. The confidence interval ranged from –0.53 to –0.18.

The within-study variance was not significant (*p* > 0.05), whereas the between-study variance was significant (*p* < 0.001). Results from the funnel plot and Egger’s regression test demonstrated no evidence of publication bias (*t*(65) = –1.523, *p* = 0.133; Fig. S2).

Moderator analysis showed that the overall effect of tDCS interventions on depressive symptoms was not moderated by either stimulation intensity (*F*(4, 62) = 2.095, *p* = 0.092) or duration (*F*(3, 63) = 1.146, *p* = 0.337). However, compared to an intensity of 1 mA, an intensity of 2 mA had a significant alleviating effect (estimate = –0.694, *p* = 0.048), suggesting a notable impact of this intensity level in reducing depressive symptoms.

#### Meta-analysis results: studies targeted F3

The multivariate meta-analysis model was performed on a subgroup of studies that targeted the left DLPFC to further examine the role of this region. This analysis included 23 studies and 50 outcomes (Table 2; Fig. S3).

The overall effect of active tDCS interventions over the left DLPFC on depressive symptoms was significant (estimate = –0.422, *t*(49) = –4.393, *p* < 0.001), with a standard error of 0.096. This indicated that the active interventions targeting this region were associated with significantly lower levels of depressive symptoms compared to the sham intervention. The confidence interval ranged from –0.616 to –0.229.

The within-study variance was not significant (*p* > 0.05), whereas the between-study variance was significant (*p* < 0.001). Results from the funnel plot and Egger’s regression test demonstrated no evidence of publication bias (*t*(48) = –1.756, *p* = 0.085; Fig. S4).

Moderator analysis showed that the overall effect of active tDCS interventions targeting the left DLPFC in reducing depressive symptoms was moderated by stimulation intensity (*F*(2, 47) = 3.696, *p* = 0.032) but not duration (*F*(2, 47) = 0.827, *p* = 0.444). Specifically, compared to an intensity of 1 mA, an intensity of 2 mA demonstrated a significant alleviating effect (estimate = –0.704, *p* = 0.037), suggesting the critical role of this intensity level in alleviating depressive symptoms, specifically in tDCS interventions targeting the left DLPFC.

### tDCS effects on anxiety symptoms

#### Overview

**Fig. 3:**
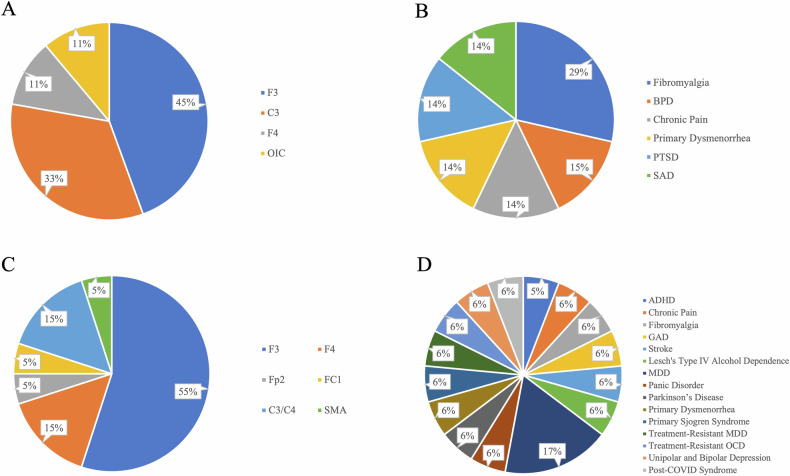
Breakdown of target regions and clinical conditions in studies with anxiety outcomes. **A** Target regions in studies with anxiety symptom alleviation; **B** Clinical conditions in studies with anxiety symptom alleviation; **C** Target regions in studies without anxiety symptom alleviation; **D** Clinical conditions in studies without anxiety symptom alleviation. (Target region abbreviation: C3, left M1; C4, right M1; F3, left DLPFC; F4, right DLPFC; FC1, left dorsal anterior cingulate cortex (dACC); Fp2, right supraorbital area; OIC, left operculo-insular cortex; SMA, supplementary motor area. Condition abbreviations: ADHD attention deficit hyperactivity disorder, BPD borderline personality disorder, GAD generalized anxiety disorder, MDD major depressive disorder, OCD obsessive-compulsive disorder, PTSD posttraumatic stress disorder, SAD social anxiety disorder).

Seven studies reported positive effects of tDCS on anxiety symptoms across clinical conditions (Table S2; Fig. 3A, B). Among these studies, four included patients with pain conditions. Four studies targeted the left DLPFC as the target region, three targeted the left M1, and one targeted the right DLPFC. All studies applied 2 mA except for one study that used 1 mA for patients with SAD [52]. Each session lasted 20 min in all studies.

Seventeen studies did not find positive effects of tDCS on anxiety symptoms (Fig. 3C, D). Among these, eleven studies stimulated the left DLPFC. Two studies employed cathodal tDCS [54, 67], which exerts inhibitory effects on the target regions. Most of these studies utilized a current intensity of 2 mA, with single sessions lasting either 20, 30, or 60 min.

#### Meta-analysis results: all studies

The multivariate meta-analysis model included 16 studies, encompassing 27 outcomes indicating levels of anxiety symptoms (Table 2; Fig. S5).

The overall effect of tDCS on anxiety symptoms was marginally significant (estimate = –0.398, *t*(26) = –2.049, *p* = 0.051), with a standard error of 0.194. The confidence interval ranged from –0.796 to 0.001.

The within-study variance was not significant (*p* > 0.05), whereas the between-study variance was significant (*p* < 0.001). Results from the funnel plot and Egger’s regression test demonstrated evidence of publication bias (*t*(25) = –2.602, *p* = 0.015; Fig. S6). As a result, the leave-out-out analysis was performed to assess the changes in the pooled estimate when studies were omitted one at a time (Fig. S7).

Moderator analysis revealed that the overall effect of tDCS interventions on anxiety symptoms was not moderated by either stimulation intensity (*F*(3, 23) = 0.483, *p* = 0.698) or duration (*F*(2, 24) = 2.767, *p* = 0.083). Notably, compared to a stimulation duration of 60 min, a duration of 20 min had a significant alleviating effect (estimate = –1.184, *p* = 0.039), suggesting a significant effect of this duration in reducing anxiety symptoms.

#### Meta-analysis results: studies targeted F3

The multivariate meta-analysis model was performed on a subgroup of studies that targeted the left DLPFC. This analysis included 9 studies and 16 outcomes (Table 2; Fig. S8).

The overall effect of tDCS over F3 on anxiety symptoms was insignificant (estimate = –0.449, *t*(15) = –1.428, *p* = 0.174), with a standard error of 0.314. This indicated that the active interventions targeting F3 and the sham interventions were associated with similar levels of anxiety symptoms. The confidence interval ranged from –1.119 to –0.221.

The within-study variance was not significant (*p* > 0.05), whereas the between-study variance was significant (*p* < 0.001). Results from the funnel plot and Egger regression demonstrated no evidence of publication bias (*t*(14) = –1.806, *p* = 0.093; Fig. S9).

Moderator analysis showed that the overall effect of tDCS interventions over F3 on anxiety symptoms was not moderated by stimulation intensity (*F*(1, 14) = 1.801, *p* = 0.201) or duration (*F*(2, 13) = 1.287, *p* = 0.309).

### tDCS effects on other affective outcomes

Table S3 presents the effects of tDCS interventions on various affective outcomes. Notably, emotional functioning was examined in eight studies, with positive effects observed for anodal tDCS over the left DLPFC, specifically on emotion regulation, executive functions related to emotional control, stress tolerance, and attentional bias towards threat-related stimuli. However, no effects were found on measures of emotional functioning without control-related factors.

### Risk of bias assessment

Figure S10 visualizes the ROB for each domain of each study. Figure S11 presents an overview of the ROB for all studies. The figures were generated using the Risk-of-bias VISualization [107]. Out of the 56 studies, 29 were judged to have a low ROB, and 27 were judged to have an unclear ROB. No study was judged to have a high ROB. Overall, the studies demonstrated high methodological quality.

## Discussion

To the best of our knowledge, this is the first systematic review and meta-analysis investigating the effects of tDCS interventions on depressive and anxiety symptoms across various clinical conditions. The findings demonstrated that tDCS interventions effectively alleviated depressive symptoms and showed promise in reducing anxiety symptoms across diverse populations. Notably, tDCS interventions targeting the left DLPFC with a 2-mA intensity significantly reduced depressive symptoms, underscoring the left DLPFC as a transdiagnostic neural mechanism underlying depressive symptoms, not only in MDD but also in other conditions. Additionally, tDCS interventions with a 20-min duration were associated with alleviated anxiety symptoms. At the clinical level, these findings inform the development of more personalized interventions based on patients’ comorbidity patterns and diagnostic profiles, thus enhancing treatment efficacy. Overall, these findings represent a crucial advancement in understanding the neuropsychological basis of depressive and anxiety symptoms and inform the development of effective tDCS interventions targeting these symptoms across diverse clinical conditions.

### tDCS alleviated depressive symptoms

#### Neural level

The findings regarding the effects of tDCS on depressive symptoms highlighted the left DLPFC as an important transdiagnostic mechanism underlying depressive symptoms across clinical conditions. This adds to the extant evidence by showing that repeated anodal tDCS over the left DLPFC alleviated depressive symptoms not only in patients diagnosed with MDD but also in patients with other clinical diagnoses (Fig. 2B).

Neuroimaging evidence has supported structural and functional differences in the left DLPFC in patients with depressive symptoms compared to healthy controls [108–111]. The left DLPFC is implicated in the altered emotion regulation in MDD [112]. Abnormal activity in the left DLPFC can lead to an impaired ability to regulate negative emotions or maintain positive emotions, resulting in persistent, more intense negative emotions [110]. Consistently, previous research employing combined tDCS and neuroimaging techniques has shed light on the mechanisms of action underlying the efficacy of tDCS interventions in reducing depressive symptoms. The treatment’s efficacy was found to be achieved through the modulation of DLPFC-involved networks, which includes inducing widespread perfusion changes, generating magnetic fields, and increasing cerebral blood flow at the left DLPFC [23, 113–115]. In addition, tDCS interventions targeting the left DLPFC demonstrated anti-depressant effects by inducing neurostructural changes at the target region [116]. These associations between neuroplasticity-related processes at the left DLPFC and the anti-depressant effect align with the findings regarding altered neurotrophic factors (such as BDNF) in MDD patients [23, 117]. The findings from this meta-analysis not only corroborate these previous studies, emphasizing the critical role of the left DLPFC in MDD but also extend the research by highlighting its importance in patients with diverse clinical diagnoses. This represents a significant step toward elucidating the neural underpinnings of depressive symptoms in various patient populations.

However, the current findings do not provide evidence of the specific neural changes associated with the alleviation of depressive symptoms by tDCS over the left DLPFC. Neuroimaging evidence has suggested activity alterations in both cortical and subcortical regions in MDD patients [118]. Based on existing neuropsychological models related to depressive symptoms, a decreased activity in frontal regions and increased activity in limbic regions may underline the altered emotion regulation in patients with depressive symptoms [119]. It has also been suggested that the imbalance between frontal and limbic regions leads to depressive symptoms [120]. Some evidence puts more emphasis on the frontal regions by suggesting a disrupted functional balance within the DLPFC in MDD patients, for example, hypoactivity in the left DLPFC and hyperactivity in the right DLPFC [121].

Although these pieces of evidence all corroborate the therapeutic efficacy of tDCS over the left DLPFC on depressive symptoms, the root causes of depressive symptoms, especially across disorders, still require examination. tDCS interventions could alleviate depressive symptoms by altering the activity of the frontal regions to exert greater control over the limbic regions, or by altering the connectivity between the frontal and limbic regions. Combining tDCS with neuroimaging techniques in future RCTs would be particularly beneficial to gain a deeper understanding of the core neural mechanisms underlying depressive symptoms in MDD and across other disorders.

#### Clinical level

The current findings demonstrate significant antidepressant effects of tDCS applied over the left DLPFC with a 2-mA intensity, providing evidence to optimize tDCS protocols not only for MDD but also across various diagnoses. Existing findings regarding the optimal intensity and duration for tDCS interventions have been inconsistent. Earlier evidence has shown that a longer stimulation duration (more than 10 min as compared to a few seconds) and a higher intensity (for example, 1 mA) could lead to longer after-effects [122, 123]. On the other hand, it has been argued that these after-effects depend more on the timing of the stimulation rather than the duration [124]. The findings of this review suggest that, compared to duration, intensity plays a more significant role in the tDCS effects on depressive symptoms, providing valuable insights into the design of treatment protocols.

Specifically, the current meta-analytic result supports that a 2-mA intensity should be strongly considered when designing tDCS protocols to reduce depressive symptoms. Although this finding was derived from participants with various clinical conditions and should be applicable across these conditions, the roles of patients’ individual characteristics and the different phenotypes of patients’ primary diagnoses need further exploration to enhance treatment planning in clinical settings. For example, the patterns observed regarding stimulation parameters suggest that a more intense tDCS protocol may be necessary to demonstrate positive effects in treatment-resistant patients. Among studies that examined depressive symptoms in treatment-resistant MDD, only Sharafi et al. [62] who administered tDCS of a current density of 0.1 mA/cm^2^ twice daily showed positive effects. Previous research has revealed that treatment-resistant and treatment-sensitive patients with MDD can be differentiated by the hypoconnectivity within the cognitive control network, which involves the DLPFC [125]. This could be the potential reason why depressive symptoms were less likely to be reduced by tDCS in treatment-resistant MDD patients and why a more intense protocol may be required. These findings underscore the significance of comprehending the subtypes of MDD psychopathology and the underlying neural differences to tailor more effective tDCS protocols based on individual characteristics. Additionally, it is crucial to investigate the biopsychosocial predictors of optimal tDCS effects for different clinical populations.

Furthermore, the findings suggest that tDCS may be a potential adjunctive treatment option for depressive symptoms in patients with other primary diagnoses. For instance, individuals diagnosed with schizophrenia may experience secondary depressive symptoms alongside their primary symptoms. If these patients are already receiving medications for their primary symptoms, they might respond more favorably to tDCS compared to traditional pharmacological treatments of depressive symptoms. However, the interaction between pharmacological treatments and tDCS is complex. Some medications, for example, benzodiazepines, have been found to reduce tDCS effects, while anti-depressants have been found to enhance tDCS effects in MDD patients [126]. Thus, for clinical practice, it would be crucial to first investigate the combined effects of tDCS and medications in various patient populations, and then explore the use of tDCS as an add-on treatment for addressing secondary depressive symptoms in these patients. Such research has the potential to enhance treatment outcomes by offering more efficient, safer, and personalized approaches to improve the emotional well-being of diverse patient groups.

### tDCS alleviated anxiety symptoms

#### Neural level

The findings regarding the effects of tDCS on anxiety symptoms suggested that tDCS is a promising tool for alleviating anxiety symptoms in patients with various clinical conditions, as the overall intervention effect was marginally significant. Although the meta-analytic result did not support that stimulating the left DLPFC drove the overall effect on anxiety symptoms, among the included studies that effectively reduced anxiety symptoms, the majority targeted the left DLPFC (Fig. 3A). Previous studies have demonstrated that the hypoactivation of the left DLPFC and hyperactivation of the right DLPFC may contribute to functional abnormalities associated with anxiety symptoms. Anodal tDCS over the left DLPFC has the potential to address this interhemispheric imbalance and subsequently alleviate anxiety symptoms. This imbalance has also been found to be implicated in depressive symptoms as discussed earlier [121]. The finding that active stimulation of the left DLPFC alleviates both symptom categories is consistent with their frequent comorbidity. The involvement of the left DLPFC as a transdiagnostic neural mechanism for both depressive and anxiety symptoms supports existing neuroimaging findings, which suggest that common prefrontal alterations may underlie the clinical similarities between MDD and anxiety disorders [127]. The current findings provide novel evidence that the left DLPFC plays a critical role in both depressive and anxiety symptoms, not only in MDD and anxiety disorders but also across other disorders.

Furthermore, tDCS over the right DLPFC has been shown to reduce anxiety symptoms in patients with BPD [103]. This finding is consistent with the results of a meta-analysis that indicated a reduction in anxiety symptoms by targeting the right DLPFC [128]. However, one study in this review [96], examining tDCS effects on both the left and right DLPFC, found no positive effects on anxiety symptoms in Parkinson’s disease patients. This inconsistency might be explained by differences in focus and methodology: targeted exclusively anxiety disorders and included both TMS and tDCS, whereas this review focused on tDCS and encompassed various diagnoses. This discrepancy highlights the need to further examine the role of the right DLPFC in reducing anxiety symptoms across various patient populations by applying diverse neuromodulatory techniques.

In addition to the DLPFC, studies that have shown positive effects on anxiety symptoms often involved stimulation of the left M1. This observation could be attributed to the fact that many of these studies focused on patients with pain conditions. Previous guidelines on tDCS have recommended anodal tDCS over the left M1 as an effective montage for reducing pain severity associated with pain conditions [27, 28]. It has been speculated that stimulating the M1 could activate neural circuits involving structures associated with the emotional component of pain processing, such as the DLPFC, thereby facilitating the inhibitory control of pain [28, 129]. It is possible that tDCS over the M1 alleviated anxiety symptoms by modulating DLPFC activity. The reduction in anxiety symptoms may be a consequence of the decreased pain severity. Further examination of the neuropsychological changes associated with tDCS over this region is essential for developing effective interventions for patients with comorbid pain and anxiety symptoms.

Compared to depressive symptoms, the findings regarding the neural underpinnings of the impact of tDCS on anxiety symptoms are less consistent. A systematic review of neuroimaging evidence has demonstrated structural or functional alterations in the frontal cortex and limbic regions in both MDD and anxiety disorders [5]. The current review’s findings, indicating that tDCS over the left DLPFC produced greater therapeutic efficacy on depressive symptoms than on anxiety symptoms, offer a novel insight suggesting that alterations in the left DLPFC region may be a more fundamental cause of depressive symptoms than anxiety symptoms. Additionally, anxiety symptoms may exhibit more distinct phenotypes across clinical conditions, making it less likely for tDCS over a specific region to generate an overall therapeutic effect on anxiety symptoms assessed by general scales. Modeling tDCS-induced electric fields in conjunction with neuroimaging techniques would be particularly valuable in understanding the changes in functional connectivity associated with the therapeutic effects of tDCS over different brain regions implicated in anxiety symptoms.

#### Clinical level

In comparison to the findings concerning depressive symptoms, tDCS interventions have shown less consistent therapeutic effects on anxiety symptoms. The overall effect was marginally significant, and neither intensity nor duration was found to be significant moderators. This finding aligns with the existing evidence-based guidelines [27, 28], which currently do not provide any recommendations for effective tDCS protocols in anxiety disorders. This disparity may be attributed to the fact that there have been fewer RCTs investigating the effects of tDCS in anxiety disorders, in contrast to those conducted for MDD. Additionally, anxiety symptoms are often less comprehensively assessed across different disorders and have less clearly defined neural underpinnings when compared to depressive symptoms. This emphasizes the necessity for larger-scale RCTs to explore the effects of tDCS on anxiety symptoms, as well as the importance of evaluating and treating anxiety symptoms to enhance the emotional well-being and quality of life in heterogeneous patient groups instead of restricting to anxiety disorders. Moreover, it is possible that studies that examined the effects of tDCS on anxiety symptoms adopted the tDCS montages that have shown positive effects on depressive symptoms due to limited evidence of effective protocols specifically on anxiety symptoms. Future trials should consider administering tDCS over other cortical regions implicated in anxiety symptoms, such as the medial PFC [130, 131], to investigate tDCS protocols with potentially greater therapeutic efficacy.

### Left DLPFC as a transdiagnostic mechanism

The findings from the current review provide support for the left DLPFC as a transdiagnostic neural mechanism underlying depressive symptoms across various clinical conditions. In addition, tDCS over the left DLPFC has also shown positive effects on other affective outcomes. Notably, tDCS over the left DLPFC specifically improved affective outcomes associated with cognitive control functions. However, no beneficial effects of anodal tDCS over the left DLPFC were observed on emotional recognition, expression, or positive and negative affect. These consistent patterns of results across diverse clinical samples reinforce the distinct role of the left DLPFC in top-down cognitive control functions [132, 133]. Repeated stimulation of this region may alter cognitive control functions, leading to enhanced control of emotional processes and altered attentional bias towards negative stimuli. These improved affective outcomes could potentially lead to the reduction of depressive symptoms. Nevertheless, it is also possible that the improved affective outcomes are downstream effects resulting from the improvement in depressive symptoms. Cognitive control functions may serve as a transdiagnostic mechanism underlying depressive symptoms across different disorders, while further investigation is needed to elucidate the relationships between improved affective outcomes and depressive symptoms, as well as the associated neuropsychological changes.

### tDCS as a transdiagnostic intervention

Although tDCS is not an FDA-approved treatment for depression or anxiety, this review found that tDCS is effective in alleviating depressive and anxiety symptoms across clinical conditions, highlighting tDCS as a transdiagnostic intervention for targeting these two symptom categories in various patient populations and the significance of further examining the treatment efficacy of tDCS in future clinical trials.

Considering the frequent co-occurrence of depressive and anxiety symptoms regardless of primary clinical diagnoses, the left DLPFC should be given priority as the target region when designing tDCS interventions to alleviate these symptoms. Moreover, in individuals at risk of developing these symptoms or comorbidity, the left DLPFC should be considered as the target region for preventive interventions. However, although the role of 2-mA-intensity tDCS interventions targeting the left DLPFC in alleviating depressive symptoms has been highlighted, the current findings still lack the specificity needed for clinicians to design more effective treatment plans. Future studies should focus on examining more specific tDCS protocols, such as current density, that demonstrate the highest therapeutic efficacy for addressing these two symptom categories across diverse patient populations. Additionally, future trials can investigate the most effective tDCS protocols for patients exhibiting different comorbidity patterns of depressive and anxiety symptoms, enabling the development of more individualized treatment plans, either as an adjuvant or stand-alone approach.

The current findings also hold significant clinical implications for patients experiencing depressive or anxiety symptoms as secondary symptoms. In cases where patients have various clinical conditions, depressive and anxiety symptoms may not receive adequate attention in their treatment plans for the primary diagnoses. tDCS intervention can serve as an effective add-on treatment to target and alleviate these affective symptoms, complementing the treatment of patients’ primary diagnoses. Exploring effective tDCS protocols for patients with different conditions to address depressive and anxiety symptoms can be particularly valuable, as it offers clinicians and patients more flexible treatment options and the potential for more effective and safer tools to enhance emotional well-being across diverse patient populations.

### Limitations and future directions

There are several limitations to consider when evaluating this current review. First, this review only included studies that examined tDCS as a stand-alone intervention, as the primary goal was to evaluate tDCS as the sole intervention for depressive and anxiety symptoms. As existing studies have reported improved clinical benefits when tDCS was combined with other treatments or interventions [126, 134, 135], future reviews should systematically investigate the augmenting effects of tDCS combined with traditional treatments to address these symptoms across conditions. This would be especially beneficial for treatment-resistant patients and those with lower socio-economic status, as tDCS is a cost-effective alternative to other brain stimulation tools and conventional treatments.

Second, this review did not conduct a quantitative analysis of the differential impacts of the timing of clinical assessment, current density, electrode size, and various sham conditions on the treatment effects. This was primarily because the current review aimed to examine the neural underpinnings of the effects of tDCS on depressive and anxiety symptoms and to inform intervention design focused on stimulation intensity and duration. However, these other factors could impact the treatment effects and are important aspects to consider in designing treatment protocols. Future studies are encouraged to quantitatively analyze these parameters to further optimize intervention design for clinical application.

Third, this review focused solely on conventional tDCS. While HD-tDCS provides more precise targeting, it was not examined due to differing stimulation protocols. Conventional tDCS, extensively studied for various applications, aligns well with our research question regarding transdiagnostic therapeutic purposes. However, our findings—that the left DLPFC is a transdiagnostic target and that intensity is crucial for treatment efficacy—could guide future HD-tDCS studies in targeting this region more precisely, potentially enhancing outcomes through more individualized interventions.

Lastly, although the PRISMA guidelines were meticulously adhered to, this review was not pre-registered.

## Conclusion

tDCS interventions targeting the left DLPFC consistently alleviate depressive symptoms and show promising effects on anxiety symptoms across various clinical conditions. This supports the role of the left DLPFC as a transdiagnostic neural mechanism underlying these symptoms, extending beyond specific disorders. The findings also highlight the critical role of stimulation intensity, specifically in the effects of tDCS on the left DLPFC, in reducing depressive symptoms. Clinically, this review demonstrates that tDCS is a viable transdiagnostic intervention applicable across clinical conditions for reducing depressive and anxiety symptoms. Tailoring interventions based on patients’ psychological and diagnostic profiles is crucial for enhancing treatment effectiveness.

### Supplementary information

Supplementary Materials: Tables and Figures

Supplementary Materials: The PRISMA Checklist
