# Supplementary material for: Evaluating the effects of tDCS on depressive and anxiety symptoms from a transdiagnostic perspective: a systematic review and meta-analysis of randomized controlled trials
Source: Transl Psychiatry. 2024 Jul 18;14:295. doi: 10.1038/s41398-024-03003-w (PMC11258305; doi:10.1038/s41398-024-03003-w)

Supplementary Materials

***Evaluating the Effects of tDCS on Depressive and Anxiety Symptoms from a Transdiagnostic Perspective: A Systematic Review and Meta-Analysis of Randomized Controlled Trials***

Esther Zhiwei Zheng ^a,b,1^, Nichol M.L. Wong ^a,b,c,^^1,*^, Angela S.Y. Yang ^a,b^, Tatia M.C. Lee ^a,b,*^

^a^ State Key Laboratory of Brain and Cognitive Sciences, The University of Hong Kong, Hong Kong

^b^ Laboratory of Neuropsychology & Human Neuroscience, The University of Hong Kong, Hong Kong

^c^ Department of Psychology, The Education University of Hong Kong, Hong Kong

^1^These authors contributed equally to this article

***Correspondence to:**

Tatia M.C. Lee, Ph.D.

Room 656, The Jockey Club Tower,

The University of Hong Kong,

Pokfulam Road, Hong Kong

E-mail: tmclee@hku.hk

Nichol M.L. Wong, Ph.D.

D1-2/F-10, Department of Psychology,

The Education University of Hong Kong,

Tai Po, Hong Kong

E-mail: nmlwong@eduhk.hk

**Table S1.** Depressive Symptoms.

| **Study** | **Sample Characteristics** | | | | | **tDCS Protocols** | | | | | | | | **Outcomes** | |
| --- | --- | --- | --- | --- | --- | --- | --- | --- | --- | --- | --- | --- | --- | --- | --- |
|  | **Diagnosis** | **N (Active/Sham)** | **% Female (Active/Sham)** | **Age (SD; Active/Sham)** | | **Montage (Anode, Cathode)** | **Intensity (mA)** | **Current Density**  **(mA/cm^2^)** | **Duration (min)** | **Session N**  **Period** | | | **Sham Condition** | **Measures** | **Improvement in Active vs. Sham (Y/N)** |
| Loo et al., 2012 | MDD | 31 / 29 | 45% / 48% | | 47.8 (12.5) / 48.6 (12.6) | F3, F8 | 2 | 0.057 | 20 | | 15  3 weeks | 1 mA current for 30s; ramp up and ramp down over 10s | | MADRS | Y |
| Sampaio-Junior et al., 2018 | Bipolar Depression | 30 / 29 | 53% / 83% | | ﻿46.2 (11.8) / ﻿45.7 (10.3) | F3, F4 | 2 | 0.08 | 30 | | 12  6 weeks | 30s of active stimulation | | MADRS | Y |
|  |  |  |  |  |  |  |  |  |  |  |  |  |  | HDRS | Y |
| Valiengo et al., 2017 | Post-Stroke Depression | 24 / 24 | 50% / 50% | | 62.2 (12.3) / 61.3 (10.6) | F3, F4 | 2 | 0.08 | 30 | | 12  6 weeks | 60s of active stimulation | | MADRS | Y |
|  |  |  |  |  |  |  |  |  |  |  |  |  |  | HDRS | Y |
| Boggio et al., 2008* | MDD | 21 / 9 / 10 | 67% / 67% / 70% | | 51.6 (7.7) / 46.3 (5.8) / 46.5 (7.1) | F3, Fp2 | 2 | 0.057 | 20 | | 10  2 weeks | Current ramped down after 30s | | HDRS | Y |
|  |  |  |  |  |  |  |  |  |  |  |  |  |  | BDI | Y |
| Fregni et al., 2006a | MDD | NR | NR | | NR | F3, Fp2 | 1 | 0.029 | 20 | | 5  5 alternate days | Stimulator turned off after a  few seconds | | HDRS | Y |
|  |  |  |  |  |  |  |  |  |  |  |  |  |  | BDI | Y |
| Salehinejad et al., 2017 | MDD | 12 / 12 | 58% / 67% | | 26.8 (7.1) / 25.5 (4.6) | F3, F4 | 2 | 0.057 | 20 | | 10  10 days | Current ramped up for 30s and then turned off | | HDRS | Y |
|  |  |  |  |  |  |  |  |  |  |  |  |  |  | BDI | Y |
| Salehinejad et al., 2015 | MDD | 15 / 15 | NR | | 28.7 (28.7) / 27.9 (27.9) | F3, F4 | 2 | 0.057 | 20 | | 10  10 days | Stimulator turned off after 30s | | HDRS | Y |
|  |  |  |  |  |  |  |  |  |  |  |  |  |  | BDI | Y |
| Fregni et al., 2006b | MDD | 9 / 9 | 55% / 67% | | 47.6 (10.4) / 45.3 (9.3) | F3, Fp2 | 1 | 0.029 | 20 | | 5  5 alternate days | Stimulator turned off after 5s | | HDRS | Y |
| Sharafi et al., 2019 | Treatment-Resistant MDD | 15 / 15 | 67% / 40% | | ﻿50.7 (10.7) / ﻿43.8 (12.6) | F3, F4 | 2 | 0.1 | 20 | | 10  5 days | 30s ramp-up of the current from 0 to 2 mA, 10s stimulation, 30s ramp-down to 0, and 20min no current | | HDRS | Y |
| Woodham et al., 2023 | MDD | 87 / 87 | 62% / 76% | | 37.1 (11.1) / 38.3 (10.9) | F3, F4 | 2 | 0.087 | 30 | | 36  10 weeks | ﻿Gradual ramp up over 120 seconds at the start and ramp down over 15 seconds at the end of each session | | MADRS  HDRS | Y  Y |
| Khedr et al., 2017 | Fibromyalgia | 18 / 18 | 94% / 94% | | 31.3 (11.0) / 33.9 (11.2) | C3, Right arm | 2 | 0.08 | 20 | | 10  2 weeks | Current applied for 30s at the beginning and end of the session | | HDRS | Y |
| da Silva et al., 2013 | Lesch’s Type IV Alcohol-Dependent Patients | 6 / 7 | 0% / 0% | | ﻿49 (40.5) / 49 (29.6) | F3, ﻿Right supradeltoid area | 2 | 0.057 | 20 | | 5  5 weeks | Stimulator gradually turned off after 20s | | HDRS | Y |
| Lisoni et al., 2020 | BPD | 15 /15 | 53% / 67% | | 38 (10.9) / 42.6 (13.6) | F4, F3 | 2 | 0.057 | 20 | | 15  3 weeks | ﻿2 mA current for 20s with a 20s ramp-up and ramp-down | | HDRS | Y |
|  |  |  |  |  |  |  |  |  |  |  |  |  |  | BDI | Y |
| Jafari et al., 2021 | ﻿SAD | 15 / 15 | 40% / 40% | | ﻿32.8 (7.5) / ﻿30.6 (7.5) | F3, Medial PFC | 1 | 0.029 | 20 | | 10  1 week | Current ramped up for 30s and then turned off | | BDI-II | Y |
|  |  | 15 / 15 | 53% / 40% | | ﻿33.7 (6.2) / 30.6 (7.5) | F3, Medial PFC | 2 | 0.057 | 20 | | 10  1 week | Current ramped up for 30s and then turned off | |  |  |
| Ahmadizadeh et al., 2019 | ﻿PTSD | 20 / 20 | 60% / 70% | | 44.5 (2.3) / 43.0 (2.4) | F3, F4 | 2 | 0.057 | 20 | | 10  2 weeks | ﻿Current ramped down after 30s | | BDI-II | Y |
| Quintiliano et al., 2022 | Chronic Kidney Disease ﻿Patients with Chronic Pain | 15 / 15 | 86% / 60% | | 51.5 (12.0) / 56.7 (13.6) | C3, Fp2 | 2 | 0.057 | 20 | | 10  NR | 30s of gradual current ramp-up and ramp-down | | BDI | Y |
| Liu et al., 2016 | Well-Controlled Temporal Lobe Epilepsy | 21 / 12 | 38% / 50% | | 43.3 (15.3) / 43.3 (15.5) | F3, Fp2 | 2 | 0.057 | 20 | | 5  5 days | ﻿Current ramped up for 30s and then ramped down | | BDI-II | Y |
|  |  |  |  | |  |  |  |  |  | |  |  | | NDDI-E | Y |
| Mariano et al., 2019 | Chronic Low Back Pain | 10 / 11 | 10% / 18% | | 65.7 (8.8) / 60.7 (11.8) | Right mastoid process, FC1 | 2 | 0.057 | ﻿20 | | 10  2 weeks | 30s ramp-up immediately followed by a 30s ramp-down, then 20min of stimulation averaging no more than 0.002 mA | | PHQ-9 | Y |
| Lisoni et al., 2024 | Schizophrenia | 25 / 25 | 8% / 36% | | 40.96 (13.37) / 44.44 (10.97) | F3, Fp2 | 2 | 0.057 | 20 | | 15  3 weeks | 2 mA current during 20s ramp-up and ramp-down periods only | | CDSS | Y |
| Samartin-Veiga et al., 2022 | Fibromyalgia | 34 / 30  33 / 30  33 / 30 | 100% / 100%  100% / 100%  100% / 100% | | 49.4 (8.8) / 50.7 (8.9)  50.6 (8.9) / 50.7 (8.9)  50.2 (8.2) / 50.7 (8.9) | C3, Fp2  F3, Fp2  OIC Multi-electrode montage** | 2  2  Differed by electrode** | 0.08  0.08  NR | 20  20  20 | | 15  3 weeks  15  3 weeks  15  3 weeks | Randomly and equitably  assigned to one of the 3 stimulation targets; ramps up and down were applied (15s each) at the beginning and end of the session; the current was 0 mA during the interval between the initial and the final ramps | | HADS | Y |
| Doruk et al., 2014 | Parkinson’s Disease | 6 / 7  5 / 7 | NR  NR | | NR  NR | F3, Fp2  F4, Fp1 | 2  2 | 0.057  0.057 | 20  20 | | 10  2 weeks  10  2 weeks | Two electrodes placed randomly over the F3 or F4 and the corresponding contralateral supraorbital area; current applied for the initial 30s ramp-up and 30s ramp-down | | BDI | Y |
|  |  |  |  | |  |  |  |  |  | |  |  | | HDRS | N |
| Huang et al., 2023 | Unipolar and Bipolar Depression | 23 / 24  23 / 24 | 74% / 83%  65% / 83% | | 26.7 (8.5) / 27.3 (5.6)  25.7 (5.4) / 27.3 (5.6) | Fp1, Fp2  F3, F4 | 2  2 | 0.08  0.08 | 20  20 | | 12  4 weeks  12  4 weeks | 30s ramp-up and 30s ramp-down stimulation, with 0 mA stimulation during the 20min duration | | MADRS  HDRS  QIDS-SR | Y  N  N |
| Chen et al., 2023 | MDD | 22 / 16  25 / 16 | 73% / 75%  68% / 75% | | 31.05 (9.73) / 27.94(9.74)  26.76 (7.01) / 27.94(9.74) | F3, Fp2  F3, Fp2 | 2  2 | NR  NR | 60  30 | | 10  2 weeks  10  2 weeks | Current rapidly ramped up to 2 mA over the first 30s and slowly ramped down over the next 30s | | HDRS | N |
| Loo et al., 2010 | MDD | 20 / 20 | 55% / 55% | | ﻿49.0 (10.0) / ﻿45.6 (12.5) | F3, ﻿F8 | 1 | 0.029 | 20 | | 5  NR | Current ramped down after 30s | | MADRS | N |
|  |  |  |  |  |  |  |  |  |  |  |  |  |  | HDRS | N |
|  |  |  |  |  |  |  |  |  |  |  |  |  |  | BDI | N |
| Vigod et al., 2019 | Pregnant Women with MDD | 10 / 10 | 100% / 100% | | 31.2 (4.0) / 33.3 (4.3) | F3, F4 | 2 | 0.057 | 30 | | 15  3 weeks | Current ramped down after 30s | | MADRS | N |
|  |  |  |  |  |  |  |  |  |  |  |  |  |  | EPDS | N |
| Blumberger et al., 2012 | Treatment-Resistant MDD | 13 / 11 | 77% / 91% | | ﻿45.3 (11.6) / ﻿49.7 (9.4) | F3, F4 | 2 | 0.057 | 20 | | 15  3 weeks | Stimulator turned off after 30s | | MADRS | N |
|  |  |  |  |  |  |  |  |  |  |  |  |  |  | HDRS | N |
|  |  |  |  |  |  |  |  |  |  |  |  |  |  | BDI | N |
| ﻿Bennabi et al., 2015 | Treatment-Resistant MDD | NR | 83.3% / 45.5% | | ﻿60.4 (12.0) / ﻿59.9 (15.4) | F3, Fp2 | 2 | 0.057 | 30 | | 10  5 days | Current gradually ramped down to 0 | | MADRS | N |
|  |  |  |  |  |  |  |  |  |  |  |  |  |  | HDRS | N |
|  |  |  |  |  |  |  |  |  |  |  |  |  |  | BDI | N |
| Loo et al., 2018 | MDD  Bipolar Depression | 42 / 42  19 / 17 | NR  NR | | ﻿48.9 (12.3) / ﻿46.7 (16.3)  49.1 (16.6) / ﻿47.7 (14.6) | F3, F8  F3, F8 | 2.5  2.5 | 0.071  0.071 | 30  30 | | 20  4 weeks  20  4 weeks | Current rapidly ramped up to 1 mA over the first 10s and slowly ramped down over the next minute; a second ramp up and down to 0.5 mA over 1 min was delivered at either 10min or 20min | | MADRS | N |
| Aksu et al., 2022 | Panic Disorder | 15 / 15 | 53% / 73% | | ﻿37.2 (12.8) / ﻿37.3 (10.4) | F3, F4 | 2 | 0.057 | 20 | | 10  2 weeks | ﻿30s ramp-up and ramp-down of current | | HDRS | N |
|  |  |  |  |  |  |  |  |  |  |  |  |  |  | BDI | N |
| Pinto et al., 2021b | Ischemic/Hemorrhagic Stroke | 31 / 29 | 19% / 34% | | ﻿45.6 (12.1) / ﻿48.1 (9.4) | Primary motor cortex (C3/C4) ﻿ipsilesional to the side of the stroke, Contralesional ﻿primary motor cortex (C3/C4) | ﻿Current ramped up across 30 to 60s to reach a target amplitude of between 2 and 3 mA | 0.08 – 0.12 | 30 | | 24  2 weeks | Stimulation stopped after 30 to 60s, after ramping up the current | | HDRS | N |
| Acler et al., 2013 | ﻿Post-Polio Syndrome | 16 / 16 | NR | | NR | Right and left pre-motor cortex (2 cm ahead C3-C4), Left shoulder | 1.5 | 0.043 | 15 | | 15  3 weeks | Current ramped down after 5s | | HDRS | N |
| de Lima et al., 2019 | ﻿GAD | 15 / 15 | 67% / 60% | | 32.1 (6.5) / 29.0 (5.1) | F3, Fp2 | 2 | 0.057 | 20 | | 5  5 days | Current turned off after 30s | | BDI | N |
| Silva et al., 2021 | ﻿﻿Treatment-Resistant OCD | 22 / 21 | 59% / 62% | | 38.4 (11.0) / 36.9 (12.2) | Left deltoid, SMA (﻿1.5 cm anteriorly to the measured location of Cz) | 2 | 0.08 | 30 | | 20  4 weeks | Stimulator turned off after 30s of active stimulation | | BDI | N |
| Fregni et al., 2006c | ﻿Fibromyalgia | 11 / 10  11 / 10 | 100% / 100%  100% / 100% | | 54.8 (9.3) / 50.8 (10.2)  54.2 (7.4) / 50.8 (10.2) | C3, Fp2  F3, Fp2 | 2  2 | 0.057  0.057 | 20  20 | | 5  5 days  5  5 days | Stimulator turned off after 30s; montage: C3, FP2 | | BDI | N |
| Benninger et al., 2010 | ﻿Parkinson’s Disease | 13 / 12 | 31% / 42% | | 63.6 (9.0) / 64.2 (8.8) | ﻿Symmetrically either over the premotor and motor  (electrode center 10 mm anterior to Cz) or prefrontal cortices  (forehead above eyebrows)***, ﻿Mastoids | 2 | 0.021 | 20 | | 8  2.5 weeks | ﻿Anode and cathode placed 1cm apart over the forehead  and 1 mA current applied for 1-2 min; two additional  electrodes placed inversely over the mastoids, not connected to the  stimulator; the current was ramped up over 10s and similarly decreased | | BDI | N |
| Leffa et al., 2022 | ADHD | 32 / 32 | 59% / 34% | | ﻿38.2 (10.3) / ﻿38.4 (9.1) | F4, F3 | 2 | 0.057 | 30 | | 28  4 weeks | 30s ramp-up (0-2 mA) stimulation followed by 30s ramp-down (2-0 mA) at the beginning, middle, and end of the application | | BDI | N |
| Maas et al., 2022 | Spinocerebellar Ataxia Type 3 | 10 / 10 | 30% / 50% | | 52.4 (10.8) / 51.4 (9.8) | ﻿Cerebellum, Right deltoid muscle | 2 | 0.057 | 20 | | 10  2 weeks | 40s of real stimulation, followed by a similar fade-out time and 1160s of continuous impedance control without any stimulation | | PHQ-9 | N |
| Fitzgerald et al., 2014 | Schizophrenia or Schizoaffective Disorder | NR | NR | | NR | F3, TP3  F3 and F4, TP3 and TP4 | 2  2 | 0.057  0.057 | ﻿20  20 | | 15  3 weeks  15  3 weeks | Ramp up of stimulation and 30s of stimulation prior to stimulation offset | | CDSS | N |
| Jeon et al., 2018 | Schizophrenia | 26 / 28 | 50% / 53% | | 40.0 (9.4) / 39.9 (12.4) | F3, F4 | 2 | 0.08 | 30 | | 10  2 weeks | Current ramped up and then down, and stimulated at 0 mA for the remainder of the simulation time | | CDSS | N |
| Palm et al., 2016b | Schizophrenia | 10 / 10 | 50% / 0% | | 38.4 (12.9) / 34.1 (10.7) | F3, Fp2 | 2 | 0.057 | 20 | | 10  2 weeks | The dual-mode tDCS device includes a novel sham mode that mimics sensory artefacts of tDCS | | CDSS | N |
| ﻿Valiengo et al., 2020 | Schizophrenia | 50 / 50 | 18% / 22% | | 34.6 (8.4) / 35.9 (10.1) | Left DLPFC (Midway FP1/F3), left TPJ (Midway T3/P3) | 2 | 0.057 | 20 | | 10  6 weeks | ﻿Ramp-up and ramp-down periods of 40s, with  a stimulation duration of 30s at 2 mA between the ramp  phases | | CDSS | N |
| Klírová et al., 2024 | Post-COVID Syndrome | 16 / 17 | 69% / 71% | | 44.4 (10.7) / 40.1 (10.2) | F3, F4 | 2 | 0.08 | 30 | | 20  4 weeks | 30s ramp-up and 30s ramp-down stimulation, followed by a 29min rest period | | PHQ-9 | N |
| Pinto et al., 2021a | Primary Sjogren Syndrome | 18 / 18 | 100% / 100% | | ﻿55.8 (8.5) / ﻿53.1 (10.3) | F4, F3 | 2 | 0.057 | 20 | | 5  1 week | ﻿Current ramped up at the beginning and ramped down at the end of the 20min period; no current applied in between the ramp-up and ramp-down | | BDI | N |

*This study has three groups; information listed in the order of active, active control, and sham control groups; active control group montage: anode - occipital cortex (midline and 2 cm above the inion), cathode - supraorbital area.

**F3, FC1, F8, FC5, C5, P3; ﻿a ground electrode was fixed to the right earlobe; F3 electrode = -0.565 mA, FC1 electrode = -0.508 mA, F8 electrode = -0.158 mA, FC5 electrode = 0.579 mA, C5 electrode = 1.144 mA, and P3 electrode = -0.492 mA.

***Stimulated a single target area during one session and alternated the position of the anode between sessions (starting with the motor area) so that each target area was stimulated four times.

Abbreviation:

ADHD, Attention Deficit Hyperactivity Disorder; ASD, Autism Spectrum Disorder; BPD, Borderline Personality Disorder; GAD, Generalized Anxiety Disorder; MDD, Major Depressive Disorder; OCD, Obsessive-Compulsive Disorder; PTSD, Posttraumatic Stress Disorder; SAD, Social Anxiety Disorder.

C3, left M1; C4, right M1; F3, left DLPFC; F4, right DLPFC; F8, right orbit; FC1, left dorsal anterior cingulate cortex (dACC); Fp2, right supraorbital area; OIC, left operculo-insular cortex; SMA, supplementary motor area; TP3/4, ﻿temporoparietal area; TPJ, temporoparietal junction.

BDI, Beck Depression Inventory; CDSS, Calgary Depression Scale for Schizophrenia; EPDS, Edinburgh Postnatal Depression Scale; HADS, ﻿Hospital Anxiety and Depression Scale; HDRS, Hamilton Depression Rating Scale; MADRS, Montgomery–Åsberg Depression Rating Scale; NDDI-E, Neurological Disorders Depression Inventory for Epilepsy; PHQ-9, Patient Health Questionnaire; QIDS-SR, Quick Inventory of Depressive Symptomatology Self Report.

NR, not reported.

**Table S2.** Anxiety Symptoms.

| **Study** | **Sample Characteristics** | | | | **tDCS Protocols** | | | | | | **Outcomes** | |
| --- | --- | --- | --- | --- | --- | --- | --- | --- | --- | --- | --- | --- |
|  | **Diagnosis** | **N (Active/Sham)** | **% Female (Active/Sham)** | **Age (SD; Active/Sham)** | **Montage (Anode, Cathode)** | **Intensity (mA)** | **Current Density**  **(mA/cm^2^)** | **Duration (min)** | **Session N**  **Period** | **Sham Condition** | **Measures** | **Improvement in Active vs. Sham (Y/N)** |
| Dutra et al., 2020 | Primary Dysmenorrhea | 13 / 13 | 100% / 100% | 26.1 (3.8) / 21.0 (2.1) | F3, Fp2 | 2 | 0.057 | 20 | 5  5 days | Current turned off after 30s | HARS | Y |
| Khedr et al., 2017 | Fibromyalgia | 18 / 18 | 94% / 94% | 31.3 (11.0) / 33.9 (11.2) | C3, Right arm | 2 | 0.08 | 20 | 10  2 weeks | Current applied for 30s at the beginning and end of the session | HARS | Y |
| Quintiliano et al., 2022 | Chronic Kidney Disease ﻿Patients with Chronic Pain | 15 / 15 | 86% / 60% | 51.5 (12.0) / 56.7 (13.6) | C3, Fp2 | 2 | 0.057 | 20 | 10  NR | 30s of gradual current ramp-up and ramp-down | HARS | Y |
| Lisoni et al., 2020 | BPD | 15 /15 | 53% / 67% | 38 (10.9) / 42.6 (13.6) | F4, F3 | 2 | 0.057 | 20 | 15  3 weeks | ﻿2 mA current for 20s with a 20s ramp-up and ramp-down | HARS | Y |
|  |  |  |  |  |  |  |  |  |  |  | IDAS – Anxiety subitem | Y |
| Ahmadizadeh et al., 2019 | ﻿PTSD | 20 / 20 | 60% / 70% | 44.5 (2.3) / 43.0 (2.4) | F3, F4 | 2 | 0.057 | 20 | 10  2 weeks | ﻿Current ramped down after 30s | BAI | Y |
| Jafari et al., 2021 | ﻿SAD | 15 / 15 | 40% / 40% | ﻿32.8 (7.5) / ﻿30.6 (7.5) | F3, Medial PFC | 1 | 0.029 | 20 | 10  1 week | Current ramped up for 30s and then turned off | LSAS | Y |
|  |  |  |  |  |  |  |  |  |  |  | PSWQ | Y |
| Samartin-Veiga et al., 2022 | Fibromyalgia | 34 / 30  33 / 30  33 / 30 | 100% / 100%  100% / 100%  100% / 100% | 49.4 (8.8) / 50.7 (8.9)  50.6 (8.9) / 50.7 (8.9)  50.2 (8.2) / 50.7 (8.9) | C3, Fp2  F3, Fp2  OIC Multi-electrode montage* | 2  2  Differed by electrode* | 0.08  0.08  NR | 20  20  20 | 15  3 weeks  15  3 weeks  15  3 weeks | Randomly and equitably  assigned to one of the 3 stimulation targets; ramps up and down were applied (15s each) at the beginning and end of the session; the current was 0 mA during the interval between the initial and the final ramps | HADS | Y |
| Chen et al., 2023 | MDD | 22 / 16  25 / 16 | 73% / 75%  68% / 75% | 31.05 (9.73) / 27.94(9.74)  26.76 (7.01) / 27.94(9.74) | F3, Fp2  F3, Fp2 | 2  2 | NR  NR | 60  30 | 10  2 weeks  10  2 weeks | Current rapidly ramped up to 2 mA over the first 30s and slowly ramped down over the next 30s | HARS | N |
| Woodham et al., 2023 | MDD | 87 / 87 | 62% / 76% | 37.1 (11.1) / 38.3 (10.9) | F3, F4 | 2 | 0.087 | 30 | 36  10 weeks | ﻿Gradual ramp up over 120 seconds at the start and ramp down over 15 seconds at the end of each session | HARS | N |
| Huang et al., 2023 | Unipolar and Bipolar Depression | 23 / 24  23 / 24 | 74% / 83%  65% / 83% | 26.7 (8.5) / 27.3 (5.6)  25.7 (5.4) / 27.3 (5.6) | Fp1, Fp2  F3, F4 | 2  2 | 0.08  0.08 | 20  20 | 12  4 weeks  12  4 weeks | 30s ramp-up and 30s ramp-down stimulation, with 0 mA stimulation during the 20min duration | HARS | N |
| de Lima et al., 2019 | ﻿GAD | 15 / 15 | 67% / 60% | 32.1 (6.5) / 29.0 (5.1) | F3, Fp2 | 2 | 0.057 | 20 | 5  5 days | Current turned off after 30s | HARS | N |
|  |  |  |  |  |  |  |  |  |  |  | BAI | N |
| Aksu et al., 2022 | Panic Disorder | 15 / 15 | 53% / 73% | ﻿37.2 (12.8) / ﻿37.3 (10.4) | F3, F4 | 2 | 0.057 | 20 | 10  2 weeks | ﻿30s ramp-up and ramp-down of current | HARS | N |
|  |  |  |  |  |  |  |  |  |  |  | PDSS | N |
| Pegado et al., 2020 | Primary Dysmenorrhea | 11 / 9 | 100% / 100% | 21.0 (2.1) / 20.6 (2.3) | C3, Fp2 | NR | NR | 20 | 5  5 days | Current turned off after 30s | HARS | N |
| da Silva et al., 2013 | Lesch’s Type IV Alcohol-Dependent Patients | 6 / 7 | 0% / 0% | ﻿49 (40.5) / 49 (29.6) | F3, ﻿Right supradeltoid area | 2 | 0.057 | 20 | 5  5 weeks | Stimulator gradually turned off after 20s | HARS | N |
| Doruk et al., 2014 | Parkinson’s Disease | 6 / 7  5 / 7 | NR  NR | NR  NR | F3, Fp2  F4, Fp1 | 2  2 | 0.057  0.057 | 20  20 | 10  2 weeks  10  2 weeks | Two electrodes placed randomly over the F3 or F4 and the corresponding contralateral supraorbital area; current applied for the initial 30s ramp-up and 30s ramp-down | HARS | N |
| Pinto et al., 2021b | Ischemic/Hemorrhagic Stroke | 31 / 29 | 19% / 34% | ﻿45.6 (12.1) / ﻿48.1 (9.4) | Primary motor cortex (C3/C4) ﻿ipsilesional to the side of the stroke, Contralesional ﻿primary motor cortex (C3/C4) | ﻿Current ramped up across 30 to 60s to reach a target amplitude of between 2 and 3 mA | 0.08 – 0.12 | 30 | 24  2 weeks | Stimulation stopped after 30 to 60s, after ramping up the current | HARS | N |
| Silva et al., 2021 | ﻿﻿Treatment-Resistant OCD | 22 / 21 | 59% / 62% | 38.4 (11.0) / 36.9 (12.2) | Left deltoid, SMA (﻿1.5 cm anteriorly to the measured location of Cz) | 2 | 0.08 | 30 | 20  4 weeks | Stimulator turned off after 30s of active stimulation | BAI | N |
| Leffa et al., 2022 | ADHD | 32 / 32 | 59% / 34% | ﻿38.2 (10.3) / ﻿38.4 (9.1) | F4, F3 | 2 | 0.057 | 30 | 28  4 weeks | 30s ramp-up (0-2 mA) stimulation followed by 30s ramp-down (2-0 mA) at the beginning, middle, and end of the application | BAI | N |
| Vigod et al., 2019 | Pregnant Women with MDD | 10 / 10 | 100% / 100% | 31.2 (4.0) / 33.3 (4.3) | F3, F4 | 2 | 0.057 | 30 | 15  3 weeks | Current ramped down after 30s | STAI | N |
| ﻿Bennabi et al., 2015 | Treatment-Resistant MDD | NR | 83.3% / 45.5% | ﻿60.4 (12.0) / ﻿59.9 (15.4) | F3, Fp2 | 2 | 0.057 | 30 | 10  5 days | Current gradually ramped down to 0 | STAI | N |
| Fregni et al., 2006c | ﻿Fibromyalgia | 11 / 10  11 / 10 | 100% / 100%  100% / 100% | 54.8 (9.3) / 50.8 (10.2)  54.2 (7.4) / 50.8 (10.2) | C3, Fp2  F3, Fp2 | 2  2 | 0.057  0.057 | 20  20 | 5  5 days  5  5 days | Stimulator turned off after 30s; montage: C3, FP2 | VAS - Anxiety | N |
| Mariano et al., 2019 | Chronic Low Back Pain | 10 / 11 | 10% / 18% | 65.7 (8.8) / 60.7 (11.8) | Right mastoid process, FC1 | 2 | 0.057 | ﻿20 | 10  2 weeks | 30s ramp-up immediately followed by a 30s ramp-down, then 20min of stimulation averaging no more than 0.002 mA | GAD-7 | N |
|  |  |  |  |  |  |  |  |  |  |  | PASS-20 | N |
| Pinto et al., 2021a | Primary Sjogren Syndrome | 18 / 18 | 100% / 100% | ﻿55.8 (8.5) / ﻿53.1 (10.3) | F4, F3 | 2 | 0.057 | 20 | 5  1 week | ﻿Current ramped up at the beginning and ramped down at the end of the 20min period; no current applied in between the ramp-up and ramp-down | VAS - Anxiety | N |
| Klírová et al., 2024 | Post-COVID Syndrome | 16 / 17 | 69% / 71% | 44.4 (10.7) / 40.1 (10.2) | F3, F4 | 2 | 0.08 | 30 | 20  4 weeks | 30s ramp-up and 30s ramp-down stimulation, followed by a 29min rest period | GAD-7 | N |

*﻿F3, FC1, F8, FC5, C5, P3; ﻿a ground electrode was fixed to the right earlobe; F3 electrode = -0.565 mA, FC1 electrode = -0.508 mA, F8 electrode = -0.158 mA, FC5 electrode = 0.579 mA, C5 electrode = 1.144 mA, and P3 electrode = -0.492 mA.

Abbreviation:

ADHD, Attention Deficit Hyperactivity Disorder; BPD, Borderline Personality Disorder; GAD, Generalized Anxiety Disorder; MDD, Major Depressive Disorder; OCD, Obsessive-Compulsive Disorder; PTSD, Posttraumatic Stress Disorder; SAD, Social Anxiety Disorder.

C3, left M1; C4, right M1; F3, left DLPFC; F4, right DLPFC; FC1, left dorsal anterior cingulate cortex (dACC); Fp1, left supraorbital area; Fp2, ﻿right supraorbital area; OIC, left operculo-insular cortex; SMA, supplementary motor area.

BAI, Beck Anxiety Inventory; GAD-7, Generalized Anxiety Disorder Scale; HARS, Hamilton Anxiety Rating Scale; IDAS, Irritability-Depression-Anxiety Scale; LSAS, Liebowitz Social Anxiety Scale; PASS, Pain Anxiety Symptoms Scale; PDSS, Panic Disorder Severity Scale; PSWQ, Penn State Worry Questionnaire; STAI, State-Trait Anxiety Inventory; VAS, Visual Analog Scale.

NR, not reported.

**Table S3.** Other Affective Outcomes.

| **Study** | **Sample Characteristics** | | | | | **tDCS Protocols** | | | | | | **Outcomes** | |
| --- | --- | --- | --- | --- | --- | --- | --- | --- | --- | --- | --- | --- | --- |
|  | **Diagnosis** | **N (Active/Sham)** | **% Female (Active/Sham)** | | **Age (SD; Active/Sham)** | **Montage (Anode, Cathode)** | **Intensity (mA)** | **Current Density**  **(mA/cm^2^)** | **Duration (min)** | **Session N**  **Period** | **Sham Condition** | **Measures** | **Improvement in Active vs. Sham (Y/N)** |
| ***Stress*** |  |  |  |  | |  |  |  |  |  |  |  |  |
| de Lima et al., 2019 | ﻿GAD | 15 / 15 | 67% / 60% | 32.1 (6.5) / 29.0 (5.1) | | F3, Fp2 | 2 | 0.057 | 20 | 5  5 days | Current turned off after 30s | LSSI | Y |
| Ahmadizadeh et al., 2019 | ﻿PTSD | 20 / 20 | 60% / 70% | 44.5 (2.3) / 43.0 (2.4) | | F3, F4 | 2 | 0.057 | 20 | 10  2 weeks | ﻿Current ramped down after 30s | PCL-5 | Y |
|  |  |  |  |  |  |  |  |  |  |  |  | PCL-5: Re-experiencing | Y |
|  |  |  |  |  |  |  |  |  |  |  |  | ﻿ PCL-5:  Negative Alterations in Cognition and Mood | Y |
|  |  |  |  |  |  |  |  |  |  |  |  | PCL-5: Hyper-arousal | Y |
|  |  |  |  |  |  |  |  |  |  |  |  | PCL-5: Avoidance | N |
| Pinto et al., 2021a | Primary Sjogren Syndrome | 18 / 18 | 100% / 100% | ﻿55.8 (8.5) / ﻿53.1 (10.3) | | F4, F3 | 2 | 0.057 | 20 | 5  1 week | ﻿Current ramped up at the beginning and ramped down at the end of the 20min period; no current applied in between the ramp-up and ramp-down | VAS - Stress | Y |
|  |  |  |  |  |  |  |  |  |  |  |  | Salivary Cortisol Dosage | N |
| ***Emotional Functioning*** |  |  |  |  | |  |  |  |  |  |  |  |  |
| Aksu et al., 2022 | Panic Disorder | 15 / 15 | 53% / 73% | ﻿37.2 (12.8) / ﻿37.3 (10.4) | | F3, F4 | 2 | 0.057 | 20 | 10  2 weeks | ﻿30s ramp-up and ramp-down of current | Emotional Dot-Probe Task | N |
|  |  |  |  |  |  |  |  |  |  |  |  | ERT | N |
| Jafari et al., 2021 | ﻿SAD | 15 / 15 | 40% / 40% | ﻿32.8 (7.5) / ﻿30.6 (7.5) | | F3, Medial PFC | 1 | 0.029 | 20 | 10  1 week | Current ramped up for 30s and then turned off | DERS | Y |
|  |  |  |  |  |  |  |  |  |  |  |  | Attentional Bias Task | Y |
| Barham et al., 2022 | ﻿ADHD | 11 / 11 | 55% / 82% | 22.5 (3.0) / 21.7 (2.6) | | F4, F3 | 2 | 0.057 | 20 | 5  5 days | 30s duration applied using the “fade-in, short stimulation, fade-out” protocol | RMET | N |
| Lisoni et al., 2020 | BPD | 15 /15 | 53% / 67% | 38 (10.9) / 42.6 (13.6) | | F4, F3 | 2 | 0.057 | 20 | 15  3 weeks | ﻿2 mA current for 20s with a 20s ramp-up and ramp-down | DERS | N |
| Molavi et al., 2020 | BPD | 16 / 16 | 50% / 44% | ﻿30.7 (5.0) / ﻿30.6 (5.9) | | F3, F4 | 2 | 0.08 | 20 | 10  2 weeks | Current ramped up and down for 1min, +30s of stimulation | ESQ – Emotional control | Y |
|  |  |  |  |  | |  |  |  |  |  |  | ESQ – Stress tolerance | Y |
|  |  |  |  |  | |  |  |  |  |  |  | ERQ – Cognitive reappraisal | Y |
|  |  |  |  |  | |  |  |  |  |  |  | ERQ – expressive suppression | N |
|  |  |  |  |  | |  |  |  |  |  |  | EPS – Compound score | Y |
|  |  |  |  |  | |  |  |  |  |  |  | EPS - Intrusion | Y |
|  |  |  |  |  | |  |  |  |  |  |  | EPS – Uncontrolled | Y |
|  |  |  |  |  | |  |  |  |  |  |  | EPS - Discordant | Y |
|  |  |  |  |  | |  |  |  |  |  |  | EPS – Suppression | N |
|  |  |  |  |  | |  |  |  |  |  |  | EPS – Lack of Attunement | N |
|  |  |  |  |  | |  |  |  |  |  |  | EPS - Disassociation | N |
|  |  |  |  |  | |  |  |  |  |  |  | EPS - Avoidance | N |
|  |  |  |  |  | |  |  |  |  |  |  | EPS - Externalized | N |
| Jeon et al., 2018 | Schizophrenia | 26 / 28 | 50% / 53% | 40.0 (9.4) / 39.9 (12.4) | | F3, F4 | 2 | 0.08 | 30 | 10  2 weeks | Current ramped up and then down, and stimulated at 0 mA for the remainder of the simulation time | MSCEIT - Managing Emotions | N |
| Smith et al., 2015 | Schizophrenia or Schizoaffective Disorder | 17 / 16 | 18% / 38% | 46.8 (11.1) / ﻿44.9 (9.2) | | F3, Fp2 | 2 | 0.39 | 20 | 5  8.7 ± 2.7 days | 40s of active stimulation | MSCEIT - Managing Emotions | N |
| Zemestani et al., 2022 | ASD | 17 / 15 | 29% / 27% | 8.0 (1.8) / 8.2 (2.5) | | F3, F4 | 1.5 | 0.06 | ﻿15 | 10  5 weeks | Current ramped up and down for 30s each, plus 30s of stimulation with 1.5 mA | ﻿ToM test interview - ToM1 (recognition of basic emotions) | N |
|  |  |  |  |  |  |  |  |  |  |  |  | ERC | Y |
|  |  |  |  |  |  |  |  |  |  |  |  | CPRS-RS: Emotional and behavioral problems | N |
| ***Mood*** |  |  |  |  | |  |  |  |  |  |  |  |  |
| Borckardt et al., 2013 | Patients Undergoing Unilateral TKA | 20 / 20 | NR | NR | | C1 or C2*, F4 | 2 | 0.125 | 20 | 4  NR | ﻿Current ramped down after 30s, ﻿stayed off for the remainder of the 20min | Mood ratings | N |
| Verveer et al., 2020 | Smokers | 35 / 36 | 54% / 47% | 21.1 (2.0) / 23.4 (6.2) | | F4, F3 | 2 | 0.057 | 13 | 6  3 days | Stimulator gradually turned off after 30s | Ecological Momentary Assessment - Mood | N |
| Maas et al., 2022 | Spinocerebellar Ataxia Type 3 | 10 / 10 | 30% / 50% | 52.4 (10.8) / 51.4 (9.8) | | ﻿ Cerebellum, Right deltoid muscle | 2 | 0.057 | 20 | 10  2 weeks | 40s of real stimulation, followed by a similar fade-out time and 1160s of continuous impedance control without any stimulation | POMS | N |
| Newstead et al., 2018 | Healthy Individuals | 10 / 11 | 60% / 45% | 20.4 (3.0) / 22.7 (5.9) | | F3, Right cerebellum | 2 | 0.08 | 12 | 3  5 days | ﻿Delivered a total electrical load of 5% of that given to the active condition; a ramp-up period of 15s (at 0.13 mA s^−1^), a plateau period of 6s, and a ramp-down period of 15s | POMS VAS | Y |
|  |  |  |  |  | |  |  |  |  |  |  | POMS VAS - ﻿Agreeableness | Y |
|  |  |  |  |  | |  |  |  |  |  |  | ﻿ POMS VAS - ﻿Clearheadedness | Y |
|  |  |  |  |  | |  |  |  |  |  |  | ﻿ POMS VAS - ﻿Composure | Y |
|  |  |  |  |  | |  |  |  |  |  |  | ﻿ POMS VAS - ﻿Elation | Y |
|  |  |  |  |  | |  |  |  |  |  |  | ﻿ POMS VAS - ﻿Confidence | Y |
|  |  |  |  |  | |  |  |  |  |  |  | ﻿ POMS VAS - ﻿Energy | Y |
| ***Affect*** |  |  |  |  | |  |  |  |  |  |  |  |  |
| de Lima et al., 2019 | ﻿GAD | 15 / 15 | 67% / 60% | 32.1 (6.5) / 29.0 (5.1) | | F3, Fp2 | 2 | 0.057 | 20 | 5  5 days | Current turned off after 30s | PANAS | N |
| Dutra et al., 2020 | Primary Dysmenorrhea | 13 / 13 | 100% / 100% | 26.1 (3.8) / 21.0 (2.1) | | F3, Fp2 | 2 | 0.057 | 20 | 5  5 days | Current turned off after 30s | PANAS - PA | N |
|  |  |  |  |  |  |  |  |  |  |  |  | PANAS - NA | N |
| Pegado et al., 2020 | Primary Dysmenorrhea | 11 / 9 | 100% / 100% | 21.0 (2.1) / 20.6 (2.3) | | C3, Fp2 | NR | NR | 20 | 5  5 days | Current turned off after 30s | PANAS | N |
| Quintiliano et al., 2022 | Chronic Kidney Disease ﻿Patients with Chronic Pain | 15 / 15 | 86% / 60% | 51.5 (12.0) / 56.7 (13.6) | | C3, Fp2 | 2 | 0.057 | 20 | 10  NR | 30s of gradual current ramp-up and ramp-down | PANAS | N |
| Shahbabaie et al., 2018 | Early-Abstinent Methamphetamine Users﻿ | 15 / 15 | 0% / 0% | NR | | F3, Right shoulder | 2 | 0.057 | 13:20:13** | 2  1 day | One electrode over F4 and the other over F3; stimulator turned off after 30s of ramping up and down, at the start of the stimulation session | PANAS | N |
|  |  | 15 / 15 | 0% / 0% | NR | | F4, Left shoulder | 2 | 0.057 | 13:20:13** | 2  1 day |  |  |  |
|  |  | 15 / 15 | 0% / 0% | NR | | F3, Right supraorbital ridge | 2 | 0.057 | 13:20:13** | 2  1 day |  |  |  |
|  |  | 15 / 15 | 0% / 0% | NR | | F4, Left supraorbital ridge | 2 | 0.057 | 13:20:13** | 2  1 day |  |  |  |
|  |  | 15 / 15 | 0% / 0% | NR | | F3, F4 | 2 | 0.057 | 13:20:13** | 2  1 day |  |  |  |

*Knee representation of the motor strip; C1 or C2 corresponding to the target knee.

**13 min followed by a 20-min rest and another 13 min of intervention.

Abbreviation:

ADHD, Attention Deficit Hyperactivity Disorder; ASD, Autism Spectrum Disorder; BPD, Borderline Personality Disorder; GAD, Generalized Anxiety Disorder; PTSD, Post-traumatic Stress Disorder; SAD, Social Anxiety Disorder; TKA, total knee arthroplasty.

C3, left M1; F3, left DLPFC; F4, right DLPFC; Fp2, ﻿right supraorbital area.

CPRS-RS, Conners’ Parent Rating Scale-Revised; DERS, Difficulties in Emotion Regulation Scale; ERQ, Emotion Regulation Questionnaire; EPS, Emotional Processing Scale; ERC, ﻿Emotion Regulation Checklist; ERT, Emotion Recognition Test; ESQ, Executive Skills Questionnaire for Adults; LSSI, Lipp's Stress Symptoms Inventory for Adults; MSCEIT, Mayer–Salovey–Caruso Emotional Intelligence Test; PANAS, Positive and Negative Affect Schedule; PCL-5, Posttraumatic Stress Disorder Checklist for DSM-5; POMS, Profile of Mood States; RMET, Reading the Mind in the Eyes Test; ToM, ﻿Theory of Mind; VAS, Visual Analog Scale.

NA, negative affect; PA, positive affect.

NR, not reported.

**Table S4.** Other Outcomes.

| **Study** | **Sample Characteristics** | | | | | **tDCS Protocols** | | | | | | **Outcomes** | |
| --- | --- | --- | --- | --- | --- | --- | --- | --- | --- | --- | --- | --- | --- |
|  | **Diagnosis** | **N (Active/Sham)** | **% Female (Active/Sham)** | **Age (SD; Active/Sham)** | | **Montage (Anode, Cathode)** | **Intensity (mA)** | **Current Density**  **(mA/cm^2^)** | **Duration (min)** | **Session N**  **Period** | **Sham Condition** | **Measures** | **Improvement in Active vs. Sham (Y/N)** |
| ***Mania*** |  |  |  | |  |  |  |  |  |  |  |  |  |
| Woodham et al., 2023 | MDD | 87 / 87 | 62% / 76% | | 37.1 (11.1) / 38.3 (10.9) | F3, F4 | 2 | 0.087 | 30 | 36  10 weeks | ﻿Gradual ramp up over 120 seconds at the start and ramp down over 15 seconds at the end of each session | YMRS | Y |
| Loo et al., 2018 | Bipolar Depression | 19 / 17 | NR | | ﻿49.1 (16.6) / ﻿47.7 (14.6) | F3, F8 | 2.5 | 0.071 | 30 | 20  4 weeks | Current rapidly ramped up to 1 mA over the first 10s and slowly ramped down over the next minute; a second ramp up and down to 0.5 mA over 1 min was delivered at either 10min or 20min | YMRS | N |
|  |  |  |  |  |  |  |  |  |  |  |  | ASRM | N |
| ***Psychotic Symptoms*** |  |  |  | |  |  |  |  |  |  | 30s of active stimulation |  |  |
| Mondino et al., 2016 | Schizophrenia | 11 / 12 | 27% / 42% | | 36.7 (9.7) / 37.3 (9.7) | Midway F3 and Fp1 (left DLPFC), Midway T3 and P3 (left TPJ) | 2 | 0.057 | ﻿20 | 10  5 days |  | PANSS – General Psychopathology | N |
|  |  |  |  |  |  |  |  |  |  |  |  | PANSS – Positive Symptoms | N |
|  |  |  |  |  |  |  |  |  |  |  |  | PANSS – Negative Symptoms | Y |
| Jeon et al., 2018 | Schizophrenia | 26 / 28 | 50% / 53% | | 40.0 (9.4) / 39.9 (12.4) | F3, F4 | 2 | 0.08 | 30 | 10  2 weeks | Current ramped up and then down, and stimulated at 0 mA for the remainder of the simulation time | PANSS (Total, positive, negative, general subscales) | N |
| Smith et al., 2015 | Schizophrenia or Schizoaffective Disorder | 17 / 16 | 18% / 38% | | 46.8 (11.1) / ﻿44.9 (9.2) | F3, Fp2 | 2 | 0.39 | 20 | 5  8.7 ± 2.7 days | 40s of active stimulation | PANSS | N |
| Fitzgerald et al., 2014 | Schizophrenia or Schizoaffective Disorder | NR | NR | | NR | F3, TP3  F3 and F4, TP3 and TP4 | 2  2 | 0.057  0.057 | ﻿20  20 | 15  3 weeks  15  3 weeks | Ramp up of stimulation and 30s of stimulation prior to stimulation offset | PANSS | N |
|  |  |  |  | |  |  |  |  |  |  |  | SANS | N |
| Palm et al., 2016b | Schizophrenia | 10 / 10 | 50% / 0% | | 38.4 (12.9) / 34.1 (10.7) | F3, Fp2 | 2 | 0.057 | 20 | 10  2 weeks | The dual-mode tDCS device includes a novel sham mode that mimics sensory artefacts of tDCS | SANS | Y |
|  |  |  |  | |  |  |  |  |  |  |  | SANS - Alogia | Y |
|  |  |  |  | |  |  |  |  |  |  |  | SANS - Affective, avolition/apathy, anhedonia/asociality, attention | N |
|  |  |  |  | |  |  |  |  |  |  |  | PANSS | Y |
|  |  |  |  | |  |  |  |  |  |  |  | PANSS – Negative Symptoms | Y |
|  |  |  |  | |  |  |  |  |  |  |  | PANSS – Depression/anxiety | Y |
|  |  |  |  | |  |  |  |  |  |  |  | PANSS – positive, cognition, excitement/anxiety | N |
| Koops et al., 2018 | ﻿Medication-Resistant Auditory Hallucinations (schizophrenia, psychosis NOS, schizoaffective disorders, affective disorder, borderline personality disorder) | 28 / 26 | 50% / 58% | | 44 (11) / 44 (12) | ﻿Left DLPFC (Midway FP1/F3), left TPJ (Midway T3/P3) | 2 | 0.057 | 20 | 10  5 days | 40s of real stimulation, after which only an impedance check (﻿current pulse of 110 μA) occurred every 550ms for the remainder of the treatment | PANSS | N |
| Brunelin et al., 2012 | Schizophrenia with Verbal Hallucinations | 15 / 15 | NR | | 40.4 (9.9) / 35.1 (7.0) | Left DLPFC (Midway FP1/F3), left TPJ (Midway T3/P3) | 2 | 0.057 | 20 | 10  5 days | 40s of real stimulation (2 mA), only a small current pulse occurred every 550ms (110 mA over 15ms) through the remainder of the 20min period | PANSS | Y |
|  |  |  |  |  |  |  |  |  |  |  |  | PANSS - Positive | N |
|  |  |  |  |  |  |  |  |  |  |  |  | PANSS - Negative | Y |
|  |  |  |  |  |  |  |  |  |  |  |  | PANSS - Depression | N |
|  |  |  |  |  |  |  |  |  |  |  |  | PANSS - Disorganization | N |
|  |  |  |  |  |  |  |  |  |  |  |  | PANSS – Grandiosity/excitement | N |
| ﻿Valiengo et al., 2020 | Schizophrenia | 50 / 50 | 18% / 22% | | 34.6 (8.4) / 35.9 (10.1) | Left DLPFC (Midway FP1/F3), left TPJ (Midway T3/P3) | 2 | 0.057 | 20 | 10  5 days | ﻿Ramp-up and ramp-down periods of 40s, with  a stimulation duration of 30s at 2 mA between the ramp  phases | PANSS | N |
|  |  |  |  | |  |  |  |  |  |  |  | PANSS – Negative Symptoms | Y |
|  |  |  |  | |  |  |  |  |  |  |  | PANSS – Factor Score for Negative Symptoms | Y |
|  |  |  |  | |  |  |  |  |  |  |  | PANSS – Positive Symptoms | N |
|  |  |  |  | |  |  |  |  |  |  |  | PANSS – General Symptoms | N |
|  |  |  |  | |  |  |  |  |  |  |  | SANS | N |

Abbreviation:

F3, left DLPFC; F4, right DLPFC; F8, right orbit; Fp1, left supraorbital area; Fp2, ﻿right supraorbital area; TPJ, temporoparietal junction; TP3/4, ﻿temporoparietal area.

ASRM, Altman Self-Rating Mania Scale; PANSS, Positive and Negative Syndrome Scale; SANS, Scale for the Assessment of Negative Symptoms; YMRS, Young Mania Rating Scale.

NOS, not otherwise specified; NR, not reported.

Figure S1. Forest Plot of All Studies with Depressive Outcomes.


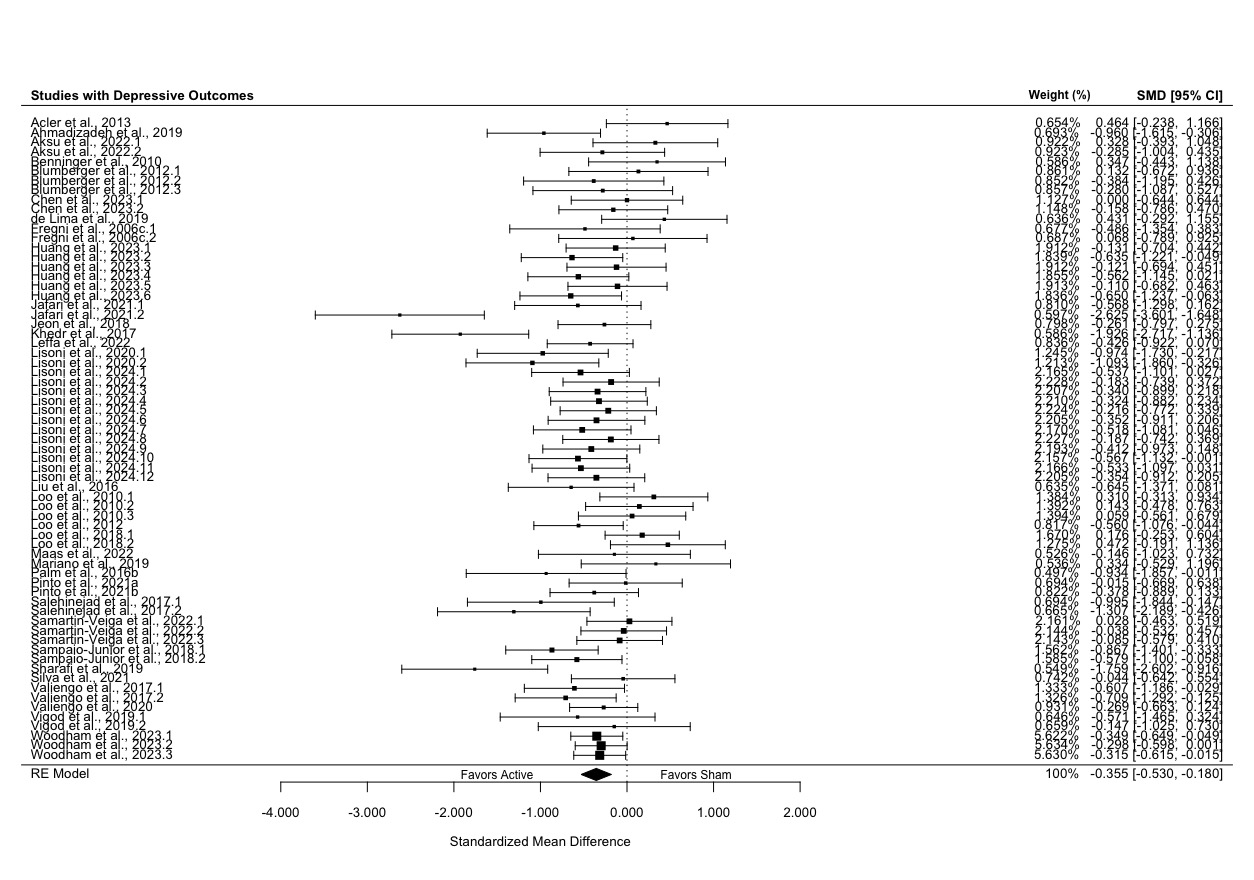


Figure S2. Funnel Plot of All Studies with Depressive Outcomes.


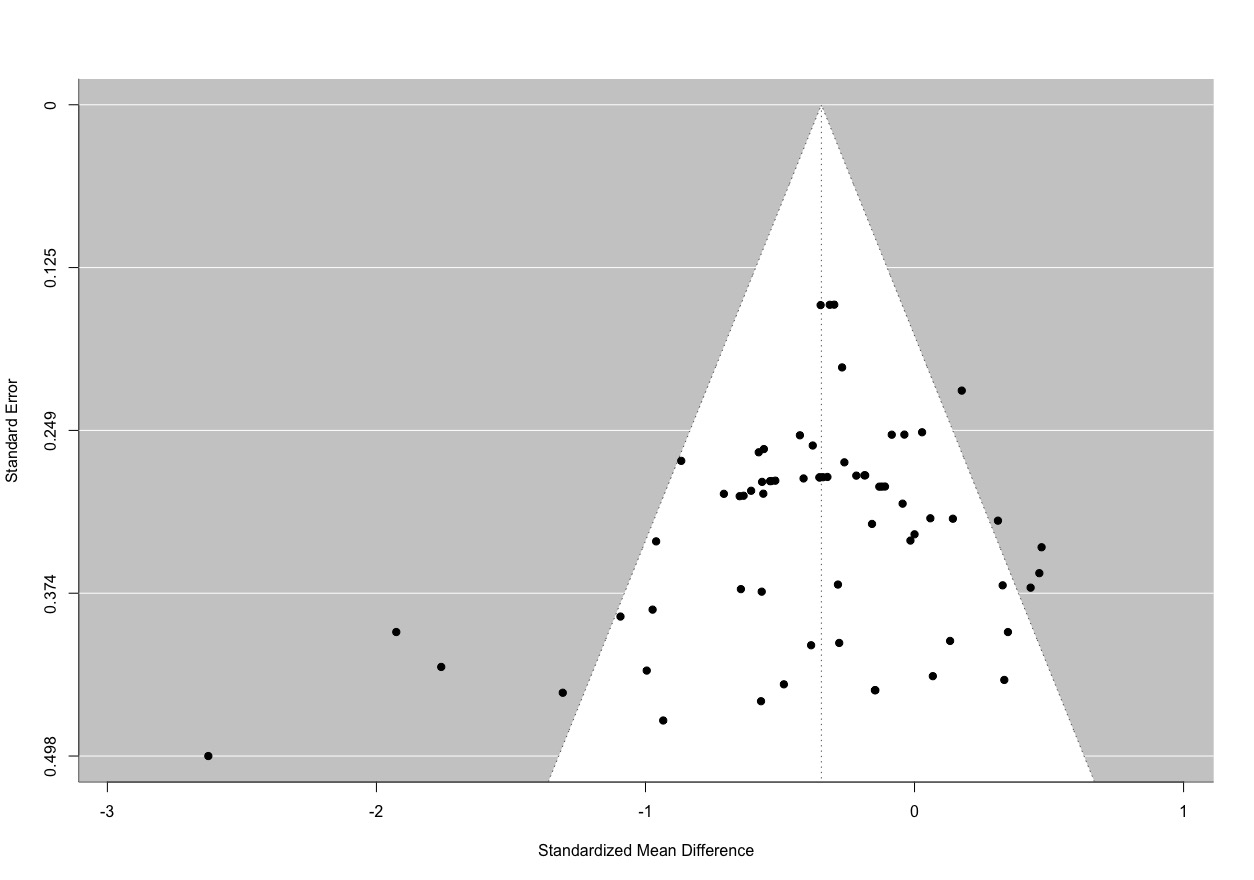


Figure S3. Forest Plot of F3 Studies with Depressive Outcomes.


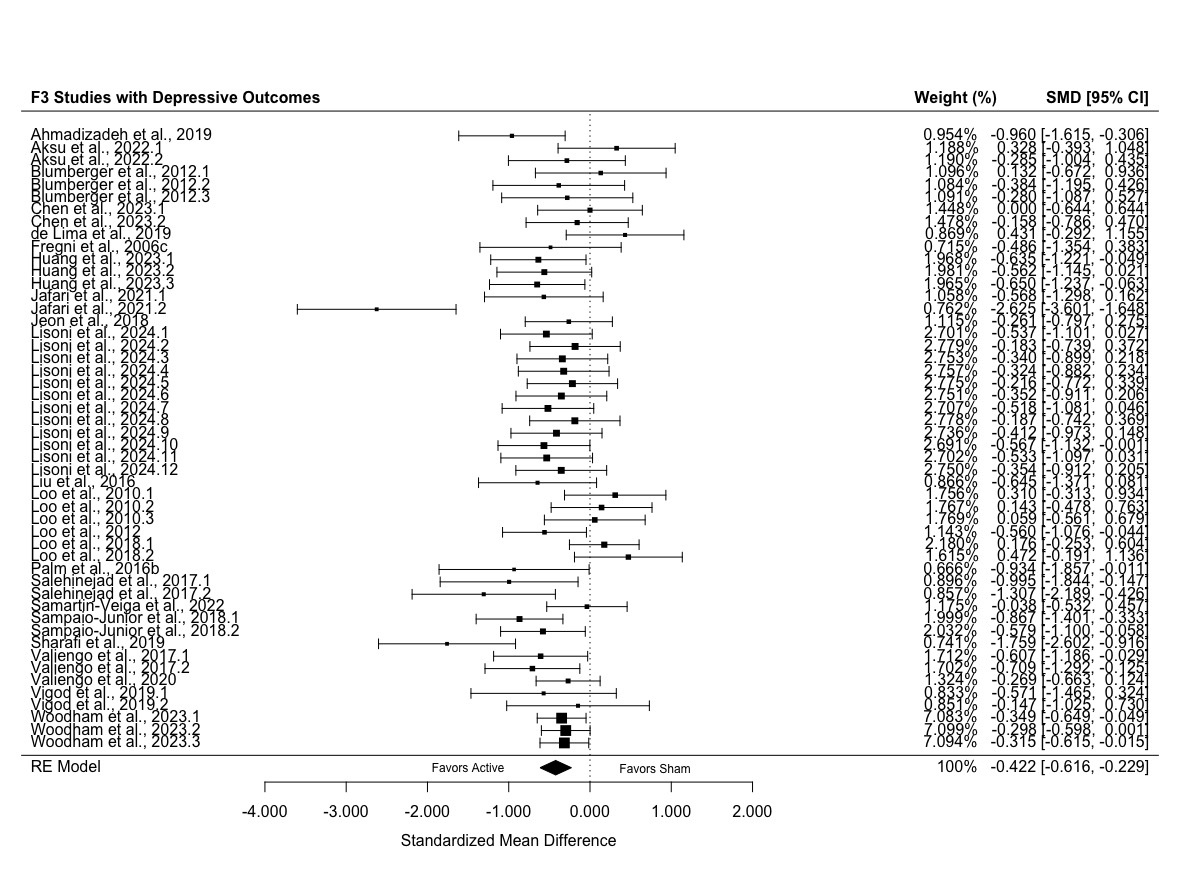


Figure S4. Funnel Plot of F3 Studies with Depressive Outcomes.


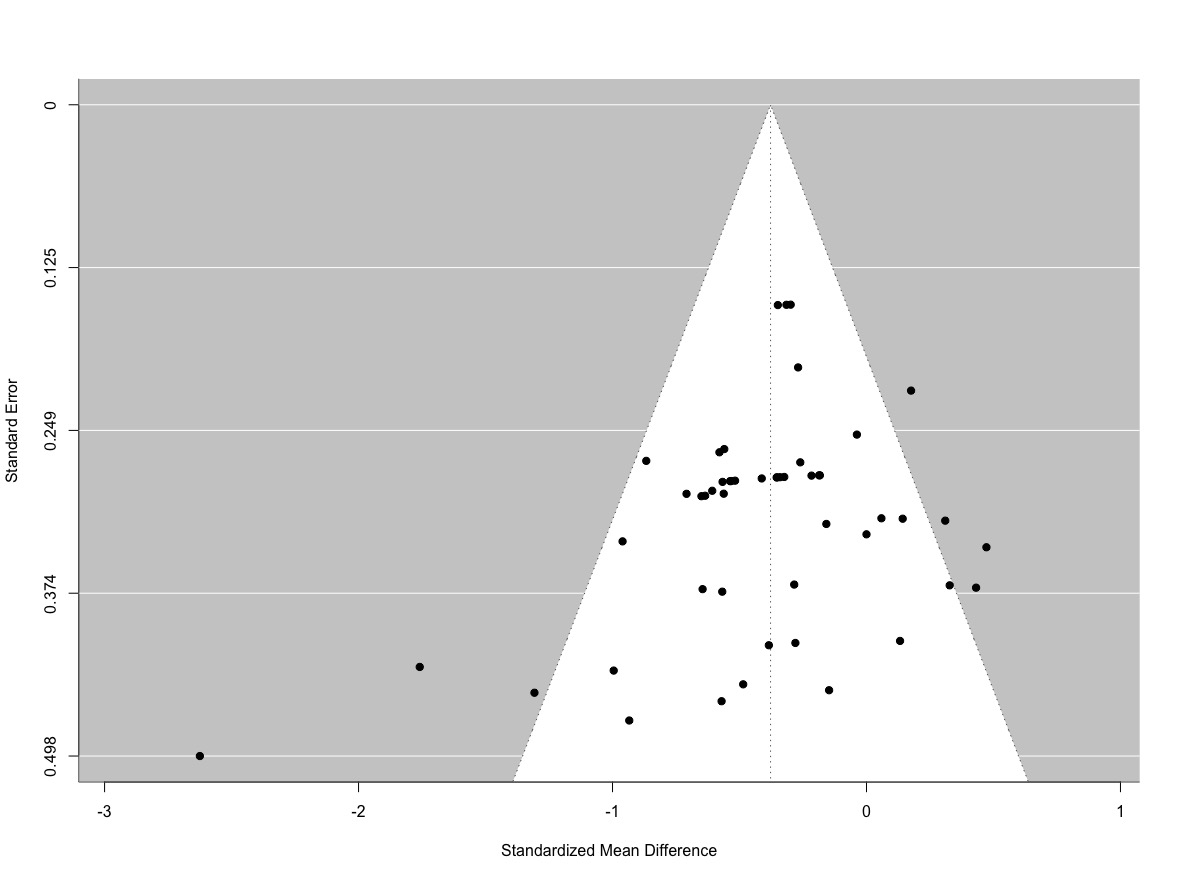


Figure S5. Forest Plot of All Studies with Anxiety Outcomes.


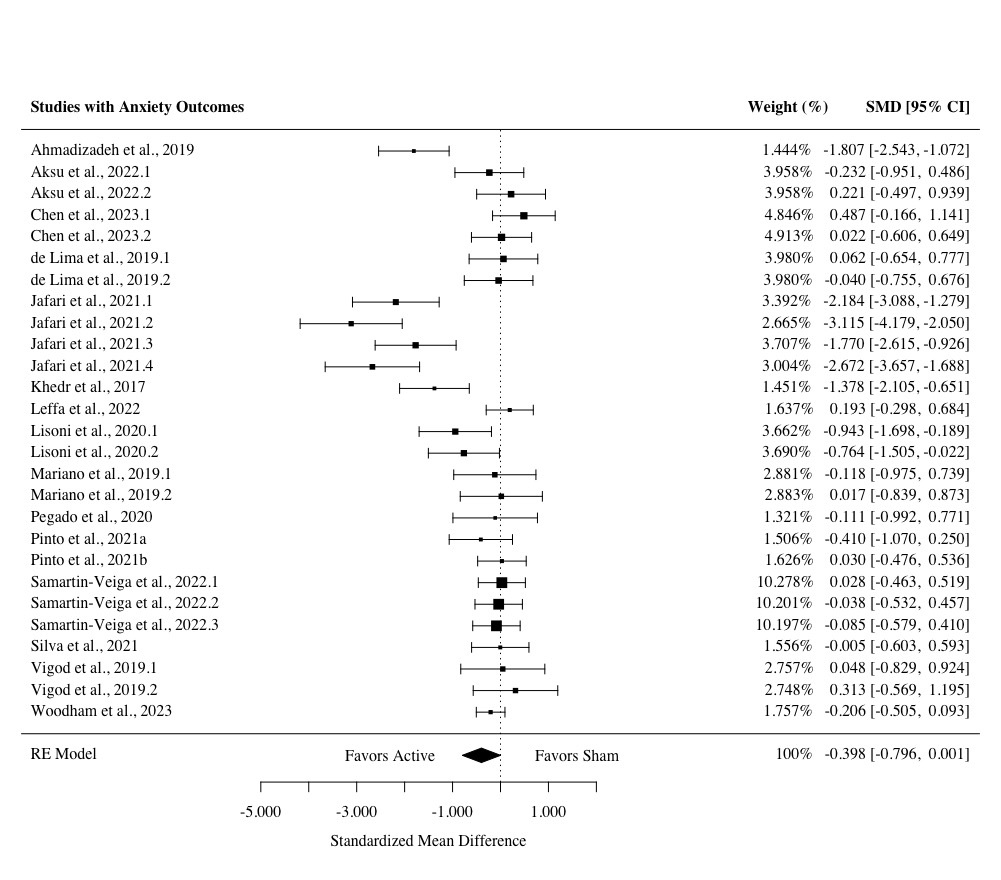


Figure S6. Funnel Plot of All Studies with Anxiety Outcomes.


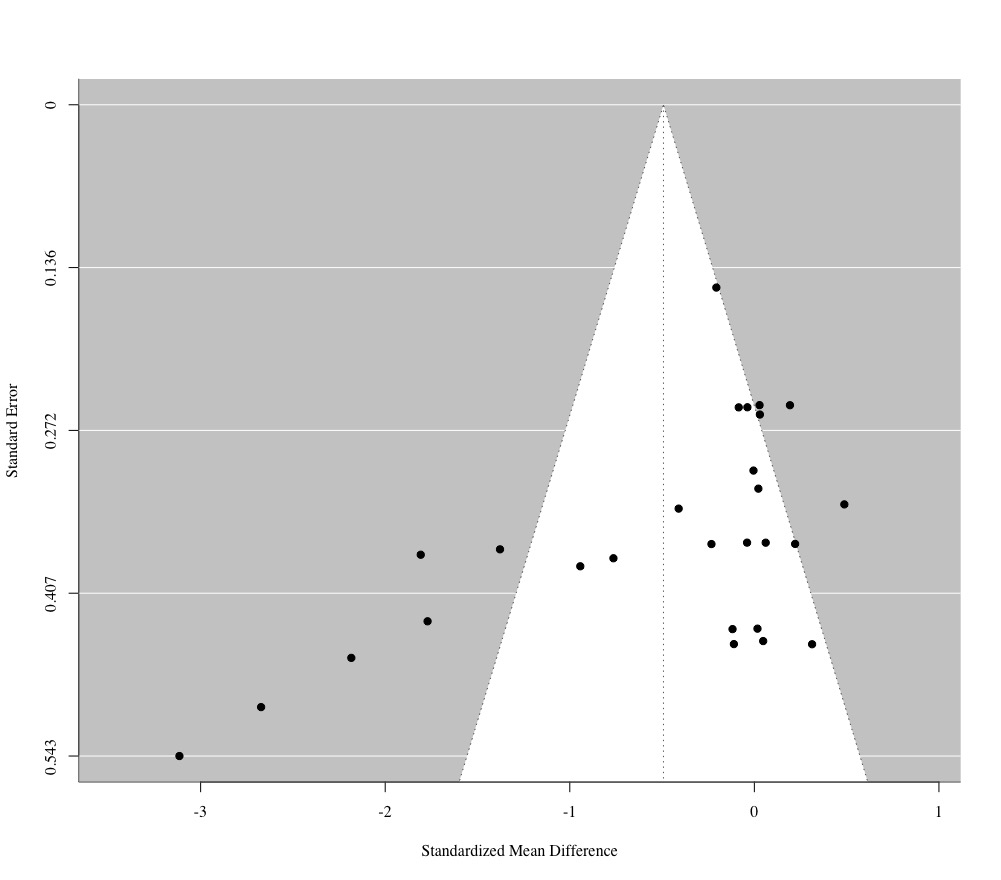


Figure S7. Forest Plot of Leave-One-Out Analysis.


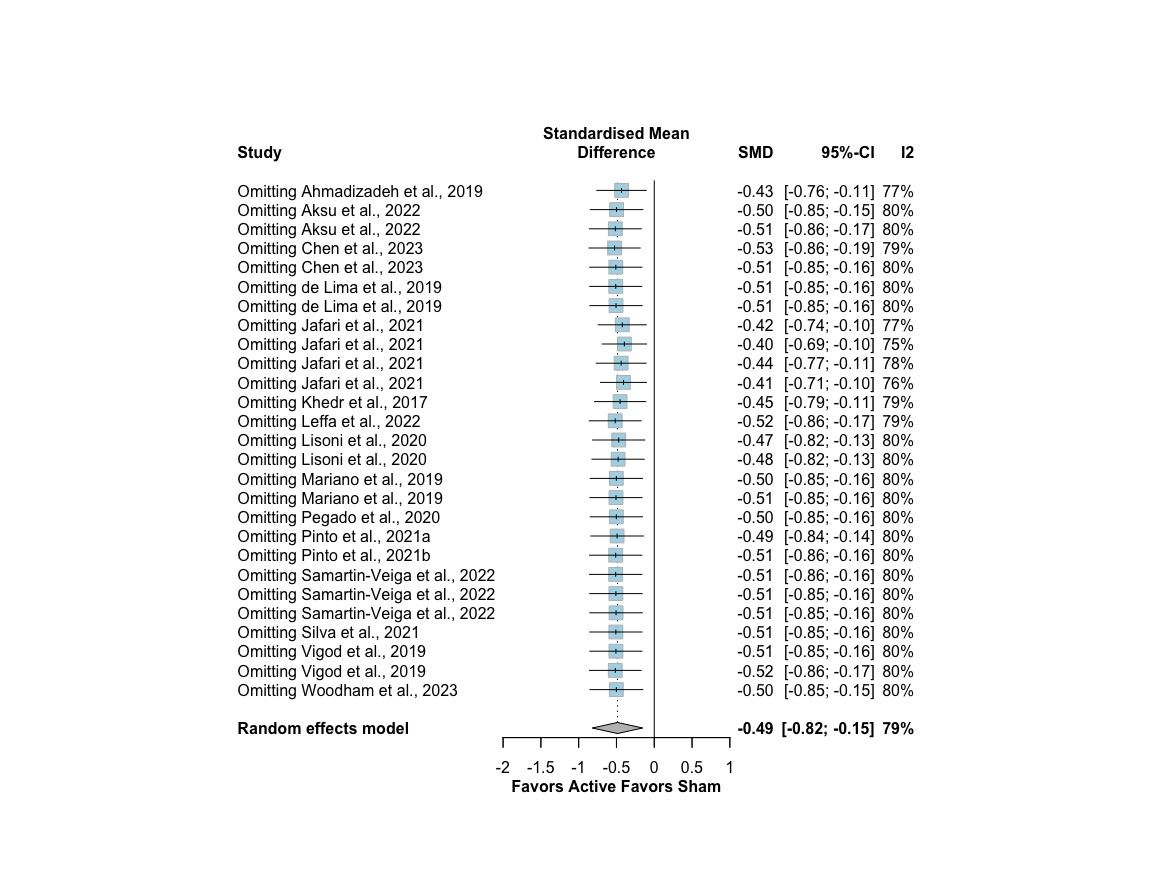


Figure S8. Forest Plot of F3 Studies with Anxiety Outcomes.


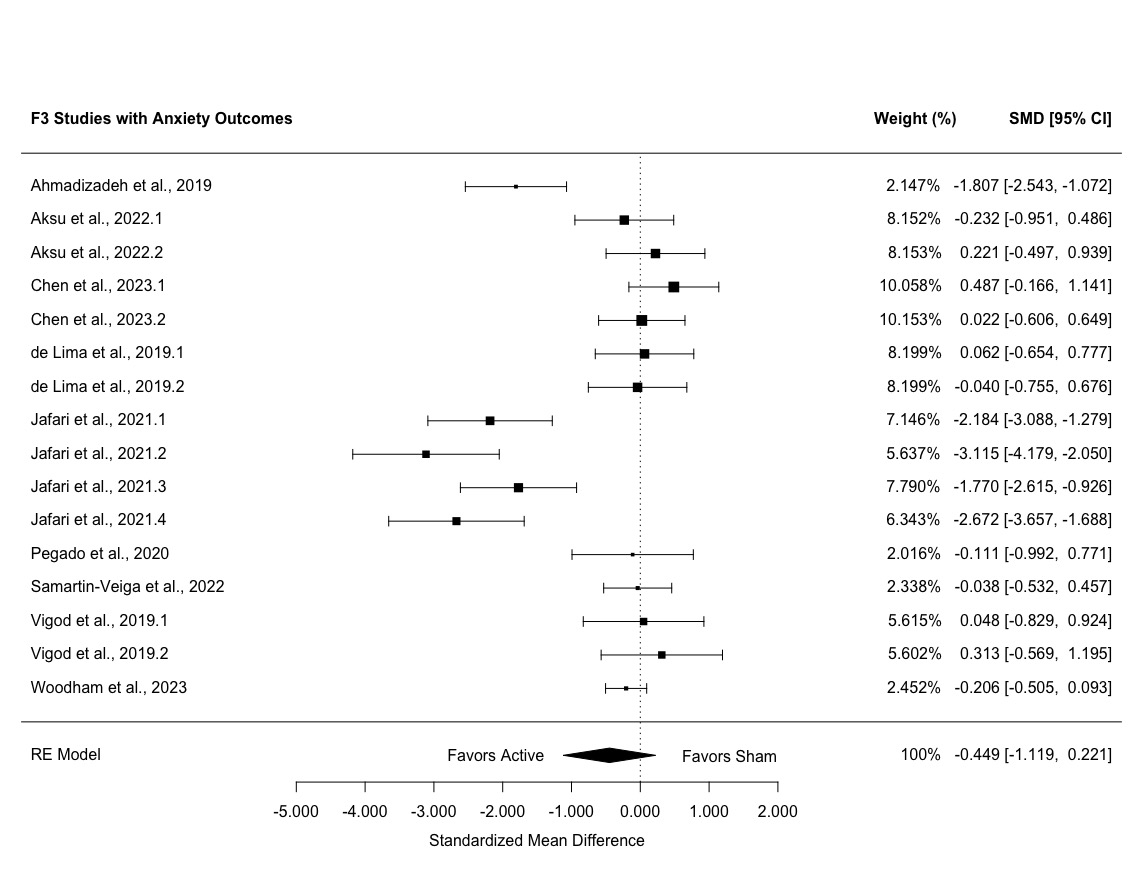


Figure S9. Funnel Plot of F3 Studies with Anxiety Outcomes.


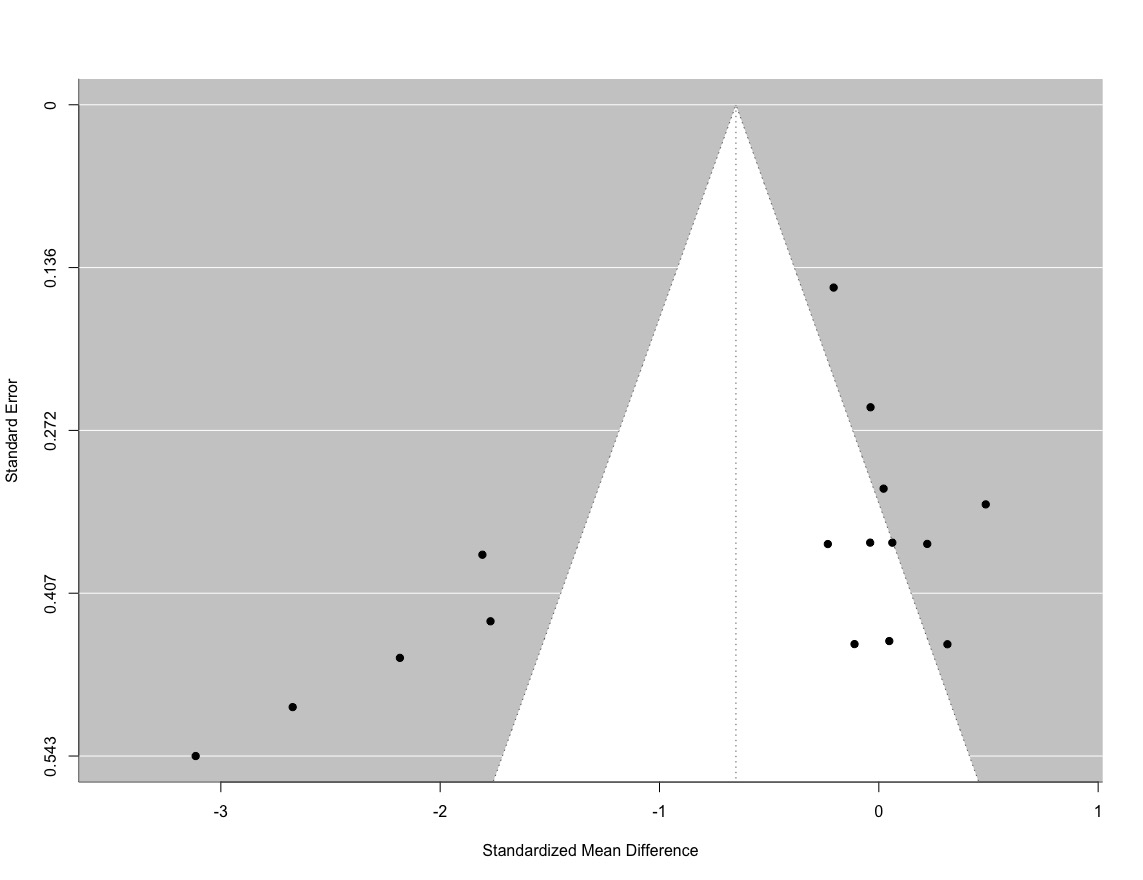


Figure S10. Risk of Bias Assessment of All Studies.


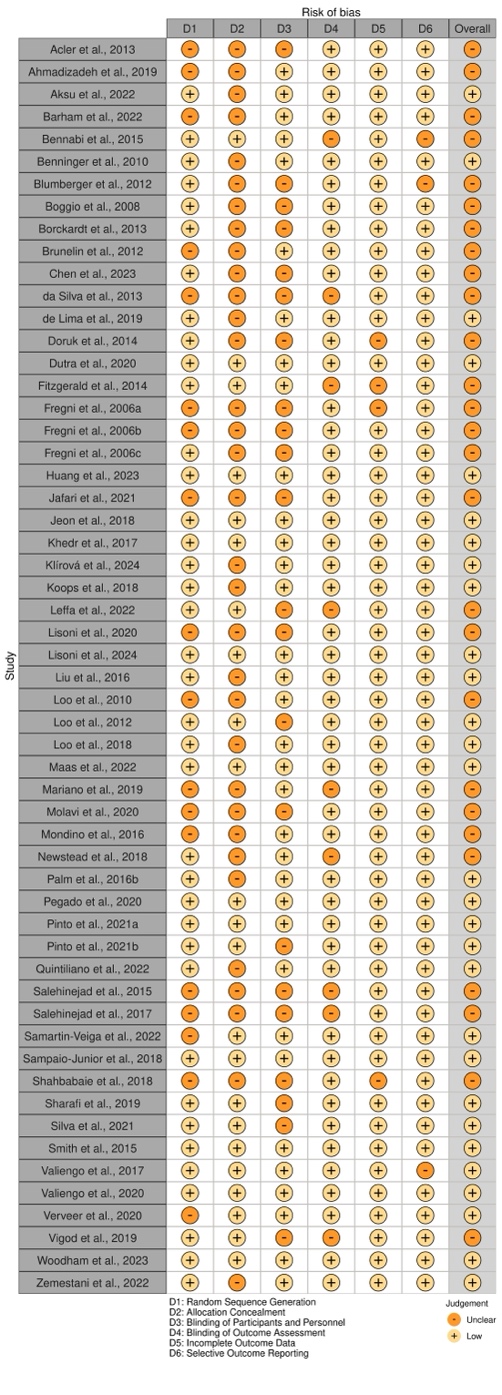


Figure S11. Risk of Bias Assessment Overview.


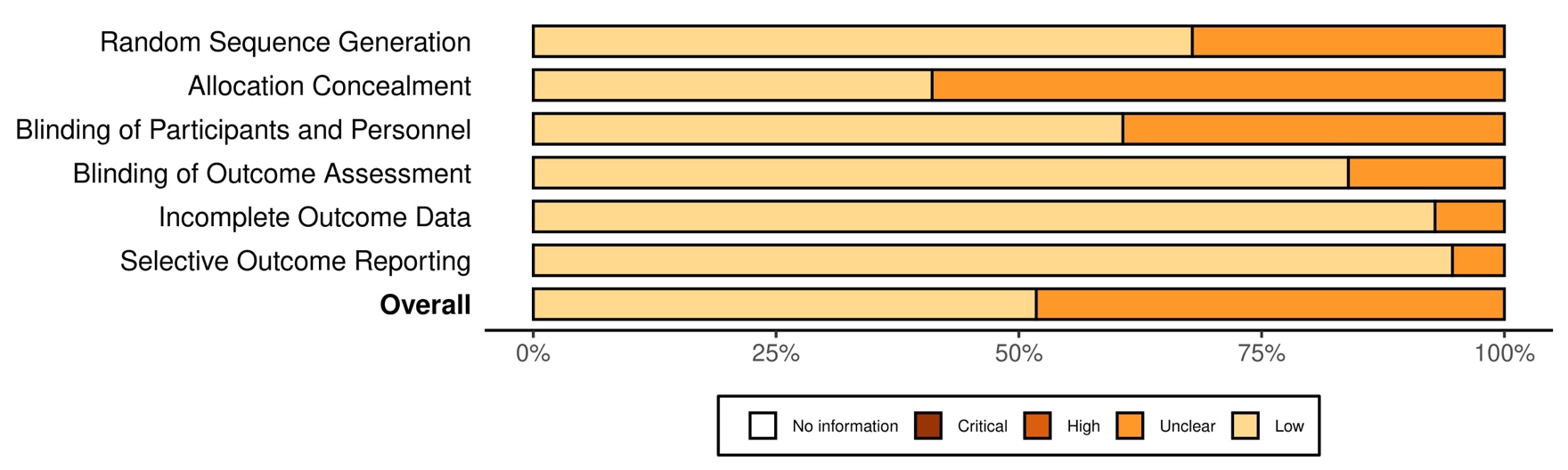

Supplement: Supplementary file 1 — Supplementary Materials: Tables and Figures [file 41398_2024_3003_MOESM1_ESM.docx]
